# Gauss curvature-based unique signatures of individual large earthquakes and its implications for customized data-driven prediction

**DOI:** 10.1038/s41598-022-12575-w

**Published:** 2022-05-23

**Authors:** In Ho Cho

**Affiliations:** CCEE Department, Iowa State University, Ames, IA 50011 USA

**Keywords:** Computational science, Natural hazards

## Abstract

Statistical descriptions of earthquakes offer important probabilistic information, and newly emerging technologies of high-precision observations and machine learning collectively advance our knowledge regarding complex earthquake behaviors. Still, there remains a formidable knowledge gap for predicting individual large earthquakes’ locations and magnitudes. Here, this study shows that the individual large earthquakes may have unique signatures that can be represented by new high-dimensional features—Gauss curvature-based coordinates. Particularly, the observed earthquake catalog data are transformed into a number of pseudo physics quantities (i.e., energy, power, vorticity, and Laplacian) which turn into smooth surface-like information via spatio-temporal convolution, giving rise to the new high-dimensional coordinates. Validations with 40-year earthquakes in the West U.S. region show that the new coordinates appear to hold uniqueness for individual large earthquakes ($M_w \ge 7.0$), and the pseudo physics quantities help identify a customized data-driven prediction model. A Bayesian evolutionary algorithm in conjunction with flexible bases can identify a data-driven model, demonstrating its promising reproduction of individual large earthquake’s location and magnitude. Results imply that an individual large earthquake can be distinguished and remembered while its best-so-far model can be customized by machine learning. This study paves a new way to data-driven automated evolution of individual earthquake prediction.

## Introduction

By virtue of the immense efforts of scientists, the statistical and probabilistic description of collective earthquakes bears meaningful fruits in understanding earthquake behaviors^1–3^ and precursory patterns for long-term earthquake forecast or small-scale events^4–6^. Statistical and geophysical knowledge are embodied by earthquake forecasting methods such as^7–9^, holding practical and scientific importance^10–15^. Combining a number of mechanics-/physics-based rules, researchers can reproduce “virtual” earthquakes on computer^16–18^. The advent of new high-precision observation technologies provides a valuable top-down viewpoint to explaining the fault, fracture, and slip behaviors^19–21^. Recently, newly emerging machine learning (ML) methods gradually play an important role in searching for hidden complex patterns of earthquakes, e.g., deep neural networks^2,22,23^, convolutional networks^24^, the gradient boosted regression trees^25^ or the random forest^26^. Despite meaningful contributions of the aforementioned approaches, we have little understanding regarding the prediction of individual large earthquakes’ location and magnitude. Collective statistical and probabilistic approaches are not directly applicable to individual earthquake prediction whereas the ML-driven exploration of earthquakes is in its infancy since the so-called black-box nature of ML methods poses challenges to the interpretability and generality. How can we fill such a formidable knowledge gap? This study seeks to address the daunting question.

This study hypothesizes that individual large earthquakes have unique signatures that can be represented by new high-dimensional features—Gauss curvature-based coordinates. In particular, this study proposes that the observed earthquake catalog data can be transformed into a number of pseudo physics quantities (i.e., energy, power, vorticity, and Laplacian) which turn into smooth surface-like information via spatio-temporal convolution, giving rise to new high-dimensional coordinates. Validations with 40-year earthquakes catalog data show that the Gauss curvature-based coordinates appear to hold uniqueness for individual large earthquakes (i.e., with moment magnitude $M_w \ge 7.0$). Also, the pseudo physics quantities may help build a customized prediction model. As an interim candidate for the model, a customized prediction model is proposed by the Bayesian evolutionary algorithm in conjunction with flexible bases functions, demonstrating a promising reproduction of individual large earthquakes’ locations and magnitudes. This study’s outcome holds an important implication for data- or ML-driven earthquake prediction research. An individual large earthquake can be uniquely distinguished and remembered by ML, and its best prediction model can be customized so that data-driven automated training and evolution can take place.

### Data preparation

Before presenting results, it is instructive to touch upon how the earthquake catalog data are processed for this research. This study collected and processed raw earthquake catalog data available in^27^ between January 1980 and October 2019 as summarized in Table S1. Without any prejudice, all the recorded earthquakes within the 40 years’ period are considered, and the total number of earthquakes amounts to 1,895,190, i.e., nearly two-million raw data points. Herein, one epoch is defined as a calendar-based, non-overlapping, one-month time frame. In detail, epoch 10000 stands for January 1980, epoch 10001 for February 1980, $\ldots$, epoch 10477 for October 2019. Due to the stochastic nature of seismicity, each epoch contains a different number to total earthquakes, e.g., epoch 10000 has 1601 data points (earthquakes) while epoch 10477 has 6886 data points. All these data sets from epoch 10000 through 10477 are made publicly available at^28^.

## Results

**Figure 1:**
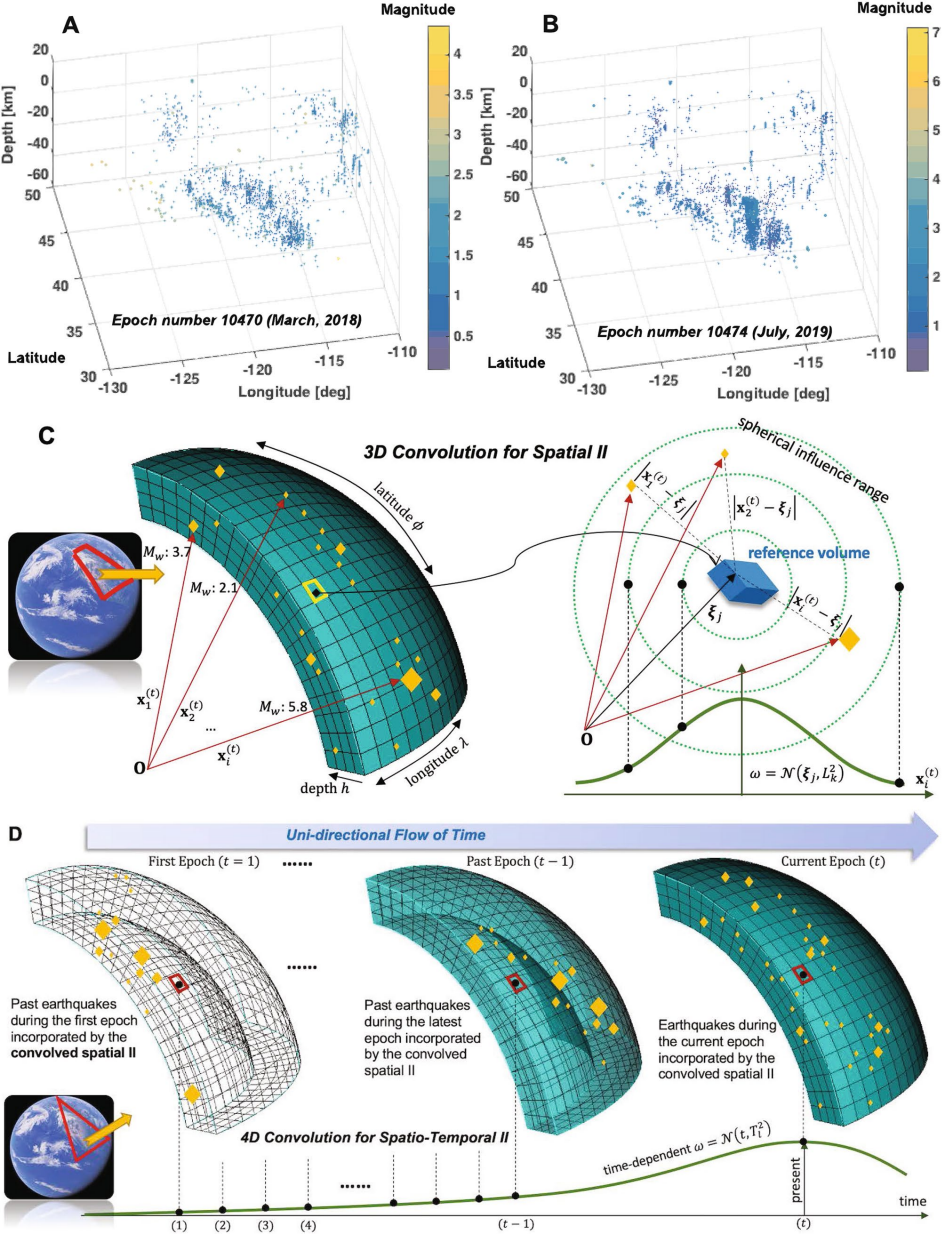
Illustration of definitions of the convolved II. **(A,B)** 3D point cloud of the recorded magnitudes of two example epochs (months) of the West region in the U.S. **(C)** Definition of the convolved spatial (3D) II. One reference volume accumulates the impacts of earthquakes during one epoch with the 3D Gaussian weights. **(D)** Convolved spatio-temporal (4D) II. Using the half Gaussian weight, all the past earthquakes are incorporated with time-decaying impacts.

This paper proposes to transform the raw earthquake catalog data into new ML-friendly scalar features (denoted as convoloved information index (II)) via spatio-temporal convolution processes, of which details and procedures are presented in “Methods” section. Figure 1 illustrates the key definitions of convolved II. Then, the convolved spatio-temporal IIs are used to engender a number of pseudo physics quantities, i.e., the released energy, power, vorticity, and Laplacian (see details in “Methods” section).

**Figure 2:**
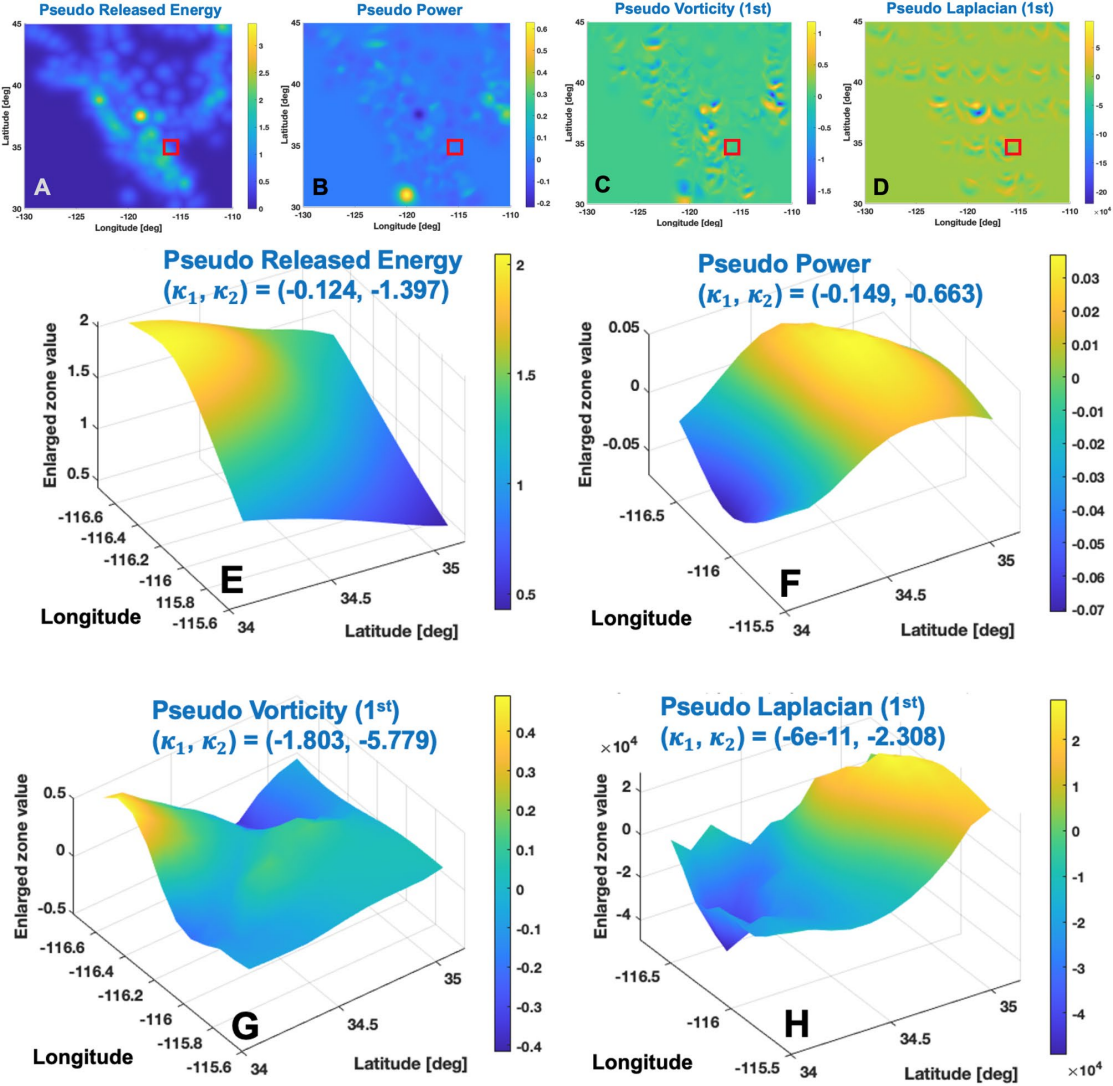
Example plots of pseudo physics quantities generated with 10 years data up to one month before the epoch 10237 (i.e., 1999/10/16): **(A–D)** The pseudo released energy ($E_r$), the pseudo power ($\partial {E_r}/\partial {t}$), the first term of pseudo vorticity $\omega _{\lambda }$, and the first term of the pseudo Laplacian $\partial ^2{E_r}/\partial {\lambda ^2}$. Red box indicate the $\pm 1 ^\circ$ zone near the peak magnitude ($M_w=7.1$) covering $(-116.25 ^\circ \pm 1 ^\circ , 34.65 ^\circ \pm 1 ^\circ )$ at depth 12.5 km; **(E–H)** Enlarged plots of the peak zone (red box in **A**–**D**) and calculated principal Gauss curvatures $(\kappa _1, \kappa _2)$.

### Gauss curvature-based unique signatures of individual large earthquakes

If there exists a unique signature before the onset of an individual large earthquake and also if the signature can be detectable and learnable, it can facilitate the prediction of an individual large event’s magnitude and location. This study seeks such unique signatures from the Gauss curvatures of the pseudo physics quantities. Since the spatio-temporal convolution process endows sufficient smoothness to the pseudo physics quantities (i.e., released energy, power, vorticity, and Laplacian), they can be regarded as a smooth surface at each depth. In lieu of simple values of the quantities, the Gauss curvature—maximum and minimum principal curvature ($\kappa _1, \kappa _2$)—can be used as informative new features (coordinates) at the event location prior to the onset of a large earthquake. To assess the uniqueness, this study calculates eight-dimensional vector $\mathbb {K}\in \mathbb {R}^8$ consisting of the principal Gauss curvatures from the four pseudo physics quantities:

1 $$\begin{aligned} \mathbb {K} :=\left( (\kappa _1, \kappa _2)_E, (\kappa _1, \kappa _2)_P, (\kappa _1, \kappa _2)_V, (\kappa _1, \kappa _2)_L)\right) \end{aligned}$$

where subscripts *E*, *P*, *V* and *L* stand for the pseudo released energy, the pseudo power, the pseudo vorticity’s first term, and the pseudo Laplacian’s first term, respectively. Eqs. 50–68 in^29^ explain the detailed calculation procedure of the Gauss curvatures using the pseudo physics quantities. Figure 2 presents the example plots of the smooth pseudo physics quantities and the associated principal Gauss curvatures near the peak magnitude zone marked by red box in Fig. 2A–D. Similar plots of the smooth pseudo physics quantities and associated principal Gauss curvatures of other large magnitude events ($M_w \ge 7.0$) are presented in Fig. S13 and Fig. S14. $\mathbb {K}$s of the eight large earthquakes of $M_w \ge 7.0$ from 1991 through 2019 in the West U.S. region are summarized in Table 1.

### Uniqueness of the Gauss curvature-based coordinates

This study hypothesizes that the Gauss curvature-based coordinates $\mathbb {K}$ of the large earthquake are unique in the past 30 years. To prove the uniqueness of $\mathbb {K}$, this study calculates comprehensive $\mathbb {K}$s of all target epochs from 10119 (i.e., December 1989) through 10477 (October 2019). To calculate a $\mathbb {K}$ of one target epoch, prior 10 years’ earthquakes data are needed (precisely, 119 months) for the spatio-temporal convolution. For instance, the $\mathbb {K}$ of target 10119 requires the information convolution using all the prior earthquakes from 10000 (January 1980) to 10118 (November 1989).

**Figure 3:**
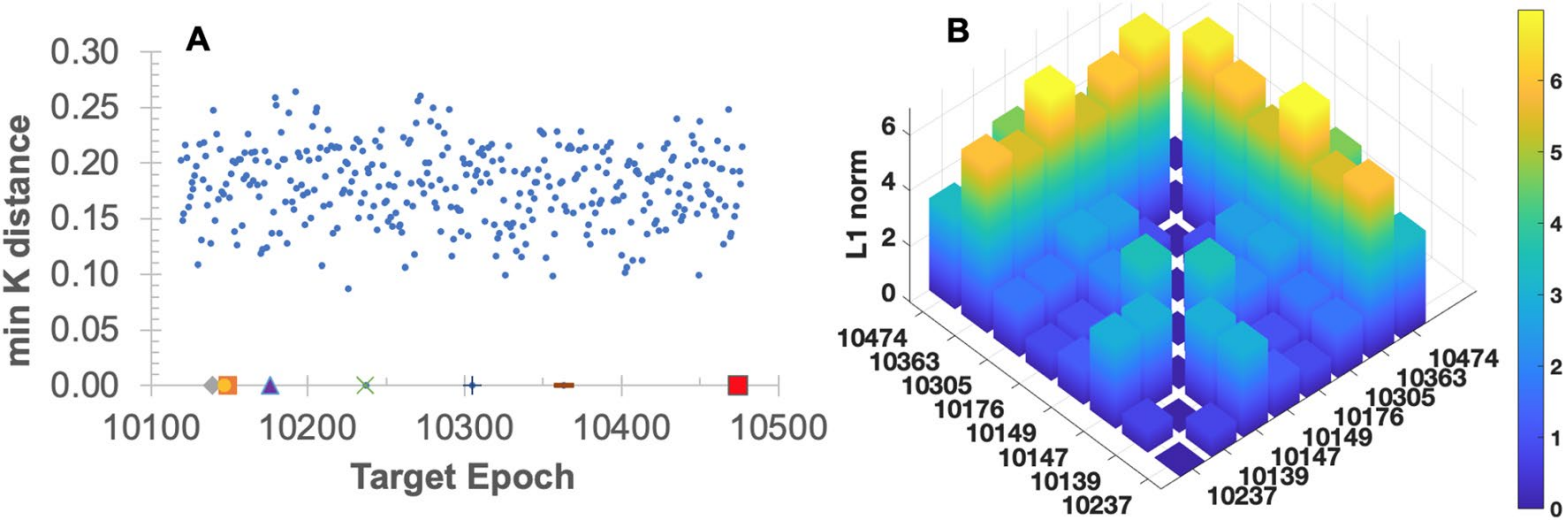
Uniqueness of large earthquakes’ Gauss curvature coordinates K of the pseudo physics quantities: **(A)** L1-norm distance of $\mathbb {K}$ of each epoch’s largest earthquake (i.e. largest event in one month) to the closest large earthquakes among the eight known earthquakes ($M_w \ge 7.0$). **(B)** L1 norm distance of $\mathbb {K}$ among the eight large earthquakes ($M_w \ge 7.0$) within 30 years. The closest two large events (epochs 10237 and 10139) are 0.73 apart in L1 norm of $\mathbb {K}$, and the farthest two large events (epochs 10147 and 10363) are 6.99 apart.

As Table 3 summarizes the algorithms, the algorithm first calculates the Gauss curvature-based coordinates $\mathbb {K}$ at the center points of the entire reference volumes in all epochs (Step-I in Table 3). At the beginning of Step-II of Table 3, $\overline{\mathbb {K}}^{(k)}, (k=1,\ldots ,8)$ at the hypocenters of the eight large earthquakes (Table 1) are calculated as a reference coordinate set. The number of reference earthquakes is 8 since only they meet the magnitude criterion ($M_w \ge 7.0$), within the 30-years time frame and in the West U.S. region under consideration. Then, a comprehensive comparison follows. In each target epoch, entire $\mathbb {K}$s of all reference volumes are compared to the eight reference earthquakes’ $\overline{\mathbb {K}}^{(k)}, (k=1,\ldots ,8)$ in terms of L1 norm. This comparison quantitatively asserts whether there is a similar $\mathbb {K}$ to any known large earthquake’s $\mathbb {K}$. Figure 3A confirms that the eight large earthquakes’ $\mathbb {K}$ appear to be unique, at least within the 40-year earthquakes used in this study. Results confirm that there are no events similar to the eight large events in terms of the Gauss curvature-based coordinates. Importantly, the L1 norm of $\mathbb {K}$ distance between any two individual large earthquakes is noticeably large (Fig. 3B), i.e., ($||\mathbb {K}-\overline{\mathbb {K}}||_{1} > 0.1$). And the large eight reference earthquakes remain uniquely distinguishable by their Gauss curvature-based coordinates. Still, it is premature to generalize this finding, calling for in-depth future investigations. However, the unique representation of individual large earthquakes via physically meaningful new coordinates may cast a new light on the “customized” prediction of an individual large earthquake as explained in the following section.

**Table 1:** Gauss curvature signatures of the eight large earthquakes, $M_w \ge 7.0$ (Subscripts *E*, *P*, *L*, and *V* correspond to the pseudo released energy, the pseudo power, the pseudo Laplacian’s first term, and the pseudo vorticity’s first term, respectively).

Target (Date)	$M_w$	$(\kappa _1, \kappa _2)_{E}$	$(\kappa _1, \kappa _2)_{P}$	$(\kappa _1, \kappa _2)_{L}$	$(\kappa _1, \kappa _2)_{V}$
10149 (1992/6)	7.3	(− 0.0159, − 2.7294)	(− 0.3504, − 2.5231)	(0, − 2.1864)	(0.5891, − 0.8117)
10139 (1991/8)	7.0	(− 0.7975, − 0.951)	(− 0.1786, − 0.418)	(0, − 0.4257)	(2.3608, − 3.7852)
10147 (1992/4)	7.2	(− 2.7935, − 6.139)	(1.1266, 0.4616)	(2.1454, 0)	(− 4.3867, − 8.9133)
10176 (1994/9)	7.0	(− 0.3237, − 1.349)	(0.5608, 0.036)	(0.3435, 0)	(0.2519, − 6.8133)
10237 (1999/10)	7.1	(− 0.1238, − 1.3966)	(− 0.1488, − 0.663)	(0, − 2.3078)	(1.8028, − 5.7792)
10305 (2005/6)	7.2	(4.2802, − 0.0505)	(0.9443, − 0.1868)	(1.1751, 0)	(0.051, − 6.7134)
10363 (2010/4)	7.2	(− 1.2262, − 3.4105)	(− 0.432, − 1.4082)	(26.5676, 0)	(8.2219, 2.2942)
10474 (2019/7)	7.1	(− 0.0471, − 1.8071)	(0.2346, − 0.0354)	(18.9517, 0)	(1.276, − 2.5747)

### Implication to customized prediction of individual large earthquakes

#### The best-so-far identified rule of magnitude prediction.

In hopes of being independent of any prior knowledge of existing magnitude prediction models^4–6,30^, or earthquake forecasting methods^5–8,10,12–15,31^, this study seeks to use a purely data-driven prediction model customized for individual large earthquakes. To this aim, this study developed a Bayesian evolutionary algorithm, of which overall architecture is illustrated in Fig. S15. The central notion in Fig. S15 is in alignment with the author’s recent application to the identification of hidden models behind nano-scale phenomena^32^ as well as complex heterogeneous structures^33,34^. In pursuit of the data-driven individual prediction model, many possible candidates of physics quantities are explored by the developed framework: the pseudo released energy $E_r$ and many forms of physical variants of $E_r$ including the three components of the spatial gradient vector $\nabla _{g} {E}_{r}^{(t)}$, the local maximums and minimums of $\nabla _{g} {E}_{r}^{(t)}$, the time derivative $\partial \nabla _{g} {E}_{r}^{(t)}/\partial {t}$ meaning the power, the pseudo vorticity (Eq. 12), and the pseudo Laplacian (Eq. 14). From the comparative investigations, the best-so-far prediction rule identified by the Bayesian evolutionary algorithm suggests including the following pseudo physics quantities: (1) the pseudo released energy (the corresponding best-so-far cubic regression spline (CRS) LF is denoted by $\mathcal {L}_{E}$), (2) the power, i.e., the time derivative of the pseudo released energy ($\mathcal {L}_{P}$), (3) the pseudo vorticity of the pseudo released energy flow ($\mathcal {L}_{\omega }$), and (4) the pseudo Laplacian ($\mathcal {L}_{L}$). The best-so-far rule of magnitude prediction is identified as the multiplicative combination of these CRS LFs of these physics quantities as

2 $$\begin{aligned} M_{pred}^{(t+1)}(\varvec{\xi }_j) = \mathcal {L}_{E}(E_r^{*(t)}) \mathcal {L}_{P}\left( \text {Sg}\left( e^2 \frac{\partial E_r^{*(t)}}{\partial {t}} \right) \right) \mathcal {L}_{\omega }\left( \text {Sg}\left( e^2\omega _{\lambda } \right) \right) \mathcal {L}_{L}\left( \text {Sg}\left( 10^{-4} \frac{\partial ^2 E_r^{*(t)}}{\partial {\lambda ^2}} \right) \right) \end{aligned}$$

where $$E_r^{*(t)}$$ is the best-so-far pseudo released energy at epoch *t* and at the reference volume $$\varvec{\xi }_j$$. The free parameters associated with the best-so-far CRS LFs $$\mathcal {L}_{E}, \mathcal {L}_{P}, \mathcal {L}_{\omega }$$, and $$\mathcal {L}_{L}$$ are denoted by $$\varvec{\uptheta }_{E}$$, $$\varvec{\uptheta }_{P}$$, $$\varvec{\uptheta }_{\omega }$$, and $$\varvec{\uptheta }_{L}$$, respectively. All terms of the pseudo physics quantities in Eq. (2) are with respect to the geodetic coordinate system, and the details regarding definitions and derivations are presented in “Methods” section. $$\text {Sg}(.)$$ stands for a typical sigmoid function, $$\text {Sg}(x) = 1/(1+e^{-x})$$, for brevity. The power term’s LF $$\mathcal {L}_{P}$$ uses the sigmoid function to transform $$\partial E_r^{(t)}(\varvec{\xi }_{j})/\partial {t} \in \mathbb {R}[-\infty , \infty ]$$ to $$\mathbb {R}(0,1)$$ which is compatible with the input range of CRS bases. A slightly modified sigmoid with a scaling-up factor $$e^2$$ is used since it appears to outperform against a typical sigmoid case. This scheme applies to the pseudo vorticity’s LF $$\mathcal {L}_{\omega }$$ since $$\omega _h \in \mathbb {R}[-\infty , \infty ]$$. Amongst many candidates for $$\mathcal {L}_{\omega }$$, e.g., $$\omega _{\lambda }, \omega _{\phi }, \omega _{h}, \text {or} \sqrt{\omega _{\lambda }^2 + \omega _{\phi }^2}$$, comparative investigations suggest that $$\omega _{\lambda }$$ appears to give the most plausible performance, as finally included in Eq. (2). Physically, $$\omega _{\lambda }$$ may describe the slow rotational motion of the energy flow about the longitudinal axis. This study’s training data are from the West U.S. region of which plate motions and the known major faults are roughly parallel or normal to the longitudinal axis. This coincidence may underpin the relatively important role of $$\omega _{\lambda }$$ in the identified rule of magnitude prediction. As mentioned earlier, the best-so-far prediction model in Eq. (2) includes the separate term of the pseudo Laplacian $$\frac{\partial ^2 E_r^{*(t)}}{\partial {\lambda ^2}}$$. Compared to the predictions using other separate terms, $$\frac{\partial ^2 E_r^{*(t)}}{\partial {\phi ^2}}, \frac{\partial ^2 E_r^{*(t)}}{\partial {h^2}}$$ or the resultant pseudo Laplacian $$\nabla _g^2 E_r^{(t)}(\varvec{\xi }_{j})$$ (here, subscript *g* indicates the geodetic coordinate system-based derivatives), the prediction only with $$\frac{\partial ^2 E_r^{*(t)}}{\partial {\lambda ^2}}$$ appears to outperform other cases. The scaling down factor $$10^{-4}$$ in Eq. (2) is due to the large range $$10^{4} \sim 10^{5}$$ of the calculated $$\frac{\partial ^2 E_r^{*(t)}}{\partial {\lambda ^2}}$$. This is another interesting coincidence with the best-performing role of $$\omega _{\lambda 
}$$ of the pseudo vorticity. The regional characteristics of the West U.S. plate’s motions may be the potential rationale behind this data-driven finding. Figure 2A–D present example plots of the smooth surface of the pseudo physics quantities. As expected, the distributions of the pseudo physics quantities are noticeably different from each other and preserves smoothness, which facilitates the calculation of the geometric quantities such as Gauss curvature.

#### Feasibility test results of individual large earthquake predictions

**Figure 4:**
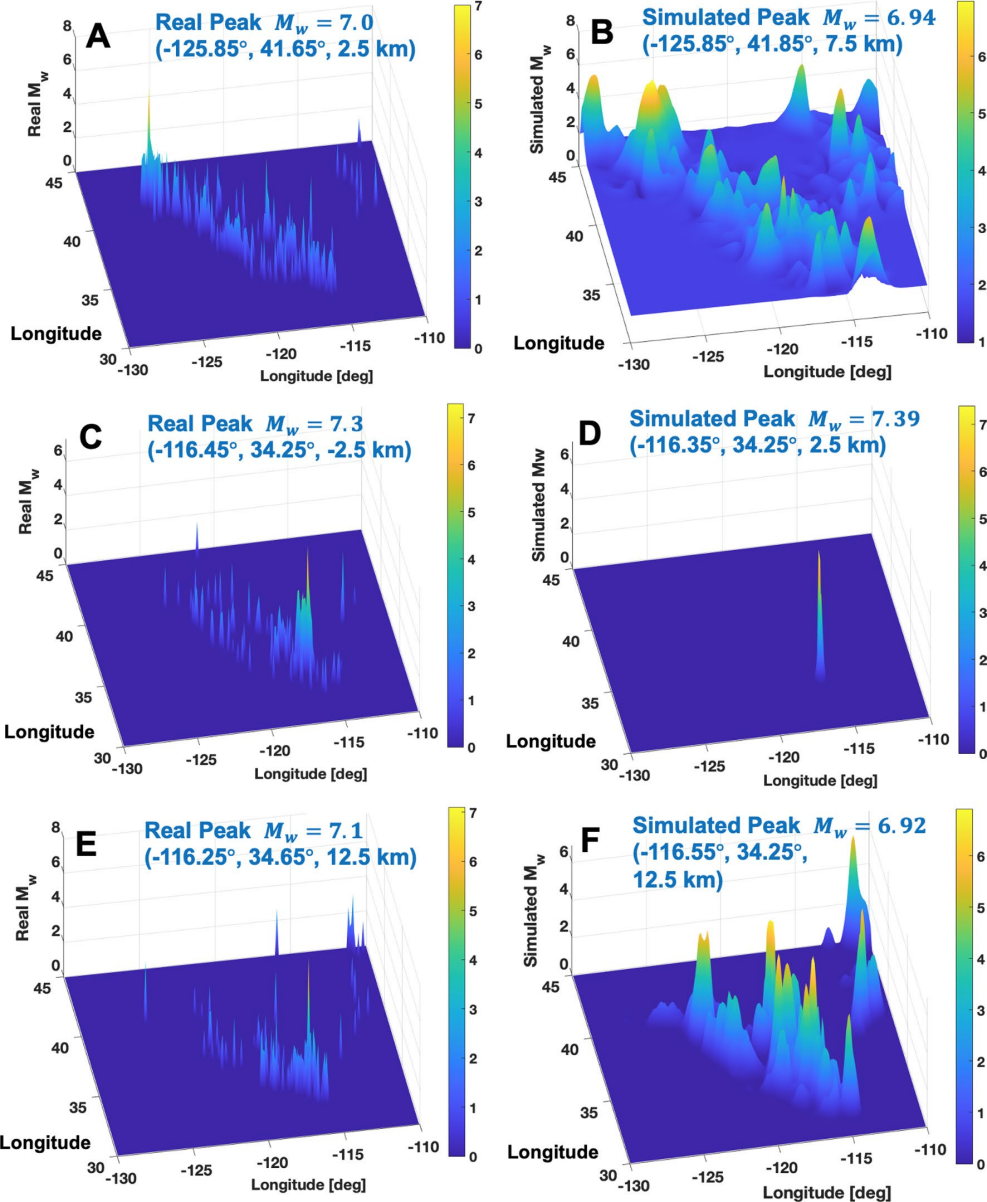
Reproduction of large magnitude events $M_w>7.0$ by using the customized ML-identified data-driven prediction model: **(A,B)** Observed real and simulated earthquake events on August 1991 (epoch 10139); **(C,D)** June 1992 (epoch 10149); **(E,F)** October 1999 (epoch 10237).

To conduct a feasibility test of the customized data-driven prediction of individual large earthquakes, this study used the ML-identified best-so-far model given in Eq. (2). It should be noted that the proposed ML-identified prediction model uses the same set of pseudo physics quantities as that is used in the generation of the Gauss curvature-based coordinates. The role of Gauss curvature-based coordinates is to offer a unique signature to individual large events, not directly to predict location and magnitude. The subsequent prediction model may use the same set of pseudo physics quantities; this study denotes such a prediction as the “associated” prediction model. Or one may choose to adopt other advanced prediction methods using other ML methods (e.g.,^22–24^), being independent of the pseudo physics quantities used in the Gauss curvature-based coordinates; this study denotes the general predictions as the “unassociated” prediction models. In this context, the present one is “associated” prediction model. The best-so-far prediction model is applied to the large reference events ($M_w \ge 7.0$) in the West U.S. region (i.e., longitude and latitude in (− 130, − 110) and (30, 45) [deg], respectively, and depth (− 5, 20) [km]) from 1990–2019. The model uses the observed 10-year data, 30 days before the event without any physics mechanisms or statistical laws. The best-so-far model appears to be successful in reproducing the next-month earthquake’s location and magnitude as shown in Fig. 4. In some cases, the ML-identified model appears to reproduce the global peak event noticeably well with little false peaks (e.g., Fig. 4C–D). In other cases, the ML-identified rules reproduce reasonably the global peak’s location and magnitude with a few false peaks (e.g., Figs. 4A,B,E,F). Table 2 summarizes the prediction results of individual eight large earthquakes using the best-so-far data-driven prediction model. Individual event’s location and magnitude are reasonably reproduced by the customized data-driven model.

**Table 2:** Individual large earthquake reproductions using the best-so-far customized data-driven models.

Target Epoch	Real	Real Peak location	Predicted	Predicted Peak location
(Year/Month)	$M_w$	$(\lambda \,[^{\circ }], \phi \,[^{\circ }], h\, \text {[km]})$	$M_w$	$(\lambda \,[^{\circ }], \phi \,[^{\circ }], h\, \text {[km]})$
10149 (1992/6)	7.3	(− 116.45, 34.25, − 2.5)	7.39	(− 116.35, 34.25, 2.5)
10139 (1991/8)	7.0	(− 125.85, 41.65, 2.5)	6.94	(− 125.85, 41.85, 7.5)
10147 (1992/4)	7.2	(− 124.25, 40.35, 7.5)	7.21	(− 124.35, 40.35, 12.5)
10176 (1994/9)	7.0	(− 126.35, 40.45, 2.5)	6.99	(− 127.75, 40.35, 17.5)
10237 (1999/10)	7.1	(− 116.25, 34.65, 12.5)	6.92	(− 116.55, 34.25, 12.5)
10305 (2005/6)	7.2	(− 125.95, 41.25, 17.5)	7.21	(− 126.25, 40.95, 7.5)
10363 (2010/4)	7.2	(− 115.25, 32.25, 7.5)	7.14	(− 115.25, 32.35, 12.5)
10474 (2019/7)	7.1	(− 117.55, 35.75, 7.5)	6.48	(− 117.75, 36.05, 12.5)

## Discussion

### A high-level analogy of the pseudo quantities to the Helmholtz’s theorem

The data-driven prediction selects out the pseudo physics quantities, i.e., pseudo released energy, power, vorticity, and Laplacian, as new important features in terms of the Gauss curvatures. The new features inherit the surface-invariant strength of the Gauss curvatures, being independent on how the surface is embedded in 3D space. It is physically clear that the released energy and its time derivative, power, appear to hold importance for large earthquakes. But, a natural question remains—why the pseudo vorticity and Laplacian quantities emerge as new important features? This section suggests a plausible mathematical answer to this question based on a high-level analogy to the Helmholtz theorem—in particular, the Helmholtz decomposition of the three-dimensional vector fields. The Helmholtz decomposition states that a smooth, differentiable, and rapidly decaying 3D vector field $\mathbf {F} \in \mathbb {R}^3$ can be decomposed into the curl-free irrotational scalar potential $\Phi \in \mathbb {R}$ and the divergence-free solenoidal vector potential $\mathbf {A}\in \mathbb {R}^3$ as

3 $$\begin{aligned} \mathbf {F}=-\nabla \Phi + \nabla \times \mathbf {A} \end{aligned}$$

With the assumption of the fast decaying $\mathbf {F}$, the potentials are given by

4 $$\begin{aligned}&\Phi (\mathbf{r})=\frac{1}{4\pi }\int _{\mathbb {R}^3} \frac{{\nabla^\prime} \cdot \mathbf {F}(\mathbf {r}')}{|\mathbf{r}-\mathbf{r^\prime}|}d{V^\prime} \end{aligned}$$

5 $$\begin{aligned}&\mathbf{A}(\mathbf{r})=\frac{1}{4\pi }\int _{\mathbb {R}^3} \frac{\nabla{^\prime} \times \mathbf{F}(\mathbf{r^\prime})}{|\mathbf{r}-\mathbf{r^\prime}|}d{V^\prime} \end{aligned}$$

where terms with $()'$ is about $\mathbf {r}'$. In principle, the Helmholtz decomposition helps elucidate complex vector fields in terms of two physically meaningful irrotational (i.e., $\Phi$) and rotational (i.e., $\mathbf {A}$) quantities. If we regard the gradient of the pseudo power as the 3D vector field by $\mathbf{{F}}(\mathbf{r})=\nabla _g \frac{\partial \textit{E}_r^{(t)}(\mathbf{r})}{\partial {t}}$, the pseudo vorticity provides a divergence-free rotational quantity by Eq. (12),

6 $$\begin{aligned} \nabla _g \times \mathbf{{F}}(\mathbf{r}) = \nabla _g \times \left( \nabla _g{ \frac{\partial \textit{E}_r^{(\textit{t})}(\mathbf{r})}{\partial {\textit{t}}}} \right) \end{aligned}$$

Here, the pseudo released energy $E_r$ is obtained by a combination of link functions by Eq. (11) which takes the convolved spatio-temporal information index $\overline{II}_{ST}$ as input. The convolution processes (Eq. 9 and Eq. 10) are re-written in a concise form as

7 $$\overline{{II}} _{{ST}} (\mathbf{r},t) = \int\limits_{{\mathbb{R} + }} {\int\limits_{{\mathbb{R}^{3} }} {\omega (\mathbf{r}^{\prime},t^{\prime})II(\mathbf{r}^{\prime},t^{\prime})dt^{\prime}d\mathbf{V}} }$$

where the Gaussian weighting function $\omega (\mathbf{r}', t')$ in the 3D space and 1D time space is $\omega \propto \exp \left( -|\mathbf{r} - \mathbf{r'}|^2 -|t-t_{past}|^2 \right)$ which is similar to the simple distance-dependent decaying function $|\mathbf{r}-\mathbf{r}'|^{-1}$ in Eq. (5). Across the given (spatial and/or temporal) domain, the proximity-dependent integration process is commonly used in the Helmholtz decomposition Eqs. (4–5) and the proposed Eq. (7). Since $\nabla \cdot (\nabla \times \mathbf{A})=0$ for $\forall \mathbf{A} \in \mathbb {R}^3$, the pseudo vorticity appears to highlight the divergence-free rotational information of the vector field under consideration.

Now, focusing on the pseudo Laplacian in Eq. (14), and regarding $\mathbf{{F}}(\mathbf{r})=\nabla _g E_r^{(t)}(\mathbf{r})$, we have

8 $$\begin{aligned} \nabla _g \cdot \mathbf{{F}}(\mathbf{r}) = \nabla _g \cdot \left( \nabla _g E_r^{(t)}(\mathbf{r})\right) \end{aligned}$$

As mentioned earlier, $E_r$ uses the Gaussian weighting function with $\exp \left( -|\mathbf{r} - \mathbf{r'}|^2 \right)$ which is comparable to the distance-dependent decaying function $|\mathbf{r}-\mathbf{r}'|^{-1}$ in Eq. (4), and also the spatial integration is commonly done in Eqs. (4), and (9). Since $\nabla \times (\nabla \phi ) = \mathbf{0}$ for $\forall {\phi \in \mathbb {R}}$, the pseudo Laplacian appears to highlight the curl-free irrotational information of the vector field under consideration. Therefore, the proposed usage of the pseudo vorticity and Laplacian can efficiently elucidate and convey the rotational and irrotational physical information of the past earthquakes, at least indirectly. It should be noted that this high-level analogy to the Helmholtz theorem is not the starting point of the adoption of the pseudo vorticity and Laplacian. Reversely, the present data-driven training and prediction process selects out the pseudo vorticity and Laplacian as relatively outstanding quantities—i.e., the inclusion of them in prediction outperforms the cases without them. The data suggests first, and the analogy to the Helmholtz theorem comes later. It appears to underpin the purely data-driven approach of this paper, in lieu of physics principle-driven approach.

**Figure 5:**
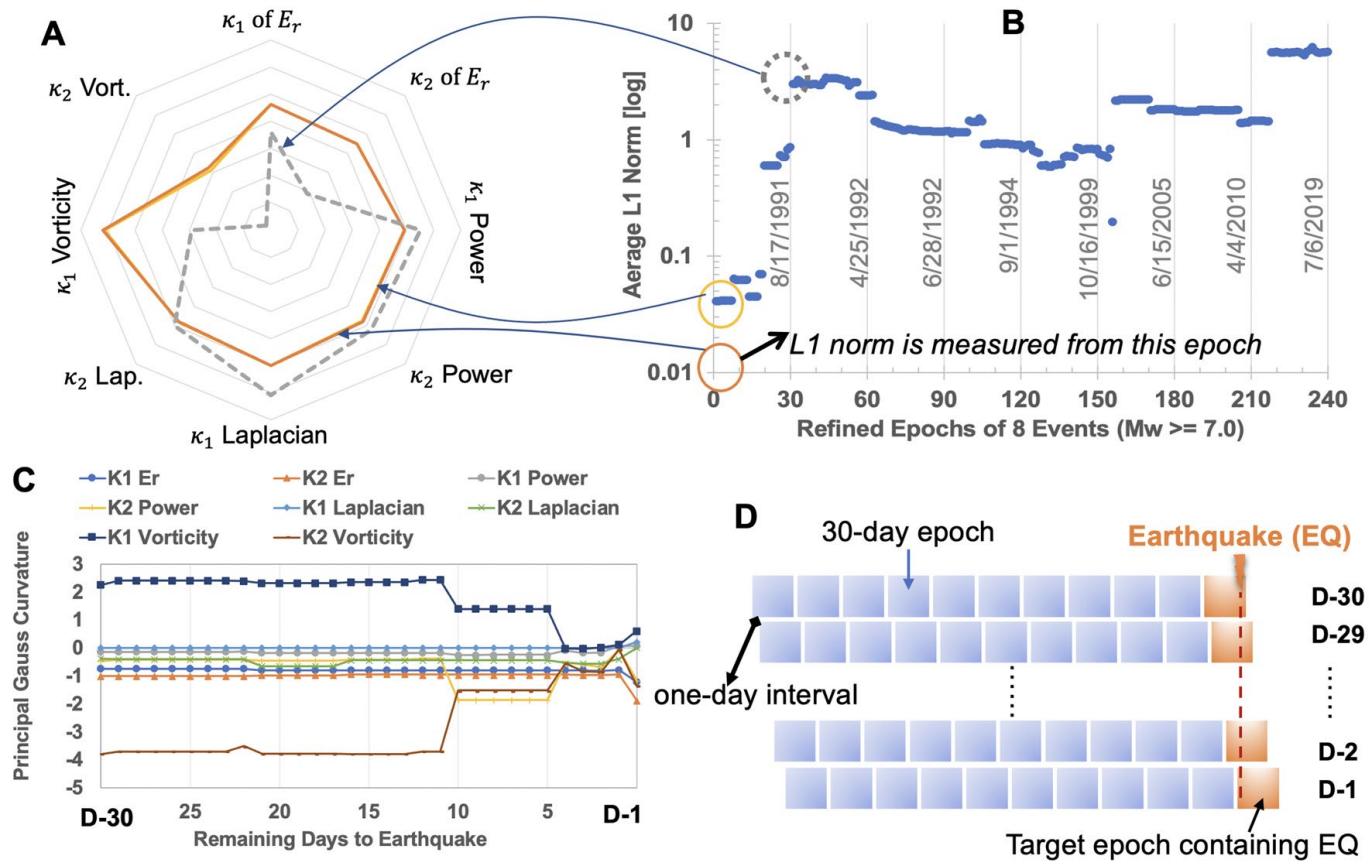
Preliminary investigation results with refined epochs with one-day interval. 10-years data one month before target earthquake (EQ) are used. (**A**) Radial plot of the principal Gauss curvatures of the pseudo physics quantities. Orange and yellow lines nearly overlap each over and indicate mutual similarity due to the same target EQ (8/17/1991) whereas dashed gray line corresponds to different target earthquake (4/25/1992) showing a noticeable discrepancy. **(B)** Average L1 norm of large earthquakes showing the relative similarity among 30 epochs before the same target EQ and the difference from epochs associated with other target EQs. **(C)** Temporal variations of 8 principal Gauss curvatures of the refined epochs before EQ (8/17/1991). **(D)** Visual explanation of the refined one-day interval of consecutive epochs.

### Remaining challenges and potential solutions by future research

It is important to remark the two challenges—data scarcity and overfitting. To overcome the data scarcity, one immediate solution would be to release the non-overlapping definition of epochs and shorten the epoch-to-epoch interval to one day from a month. One epoch is still defined as 30-day time range. As explained in Fig. 5D, if the interval between consecutive two epochs is defined by one day, we can dramatically increase the number of total epochs to 14,600 from 480 with the same 40-years earthquake catalog data. Although thorough confirmation is needed with more data sets, Fig. 5A,B support that the uniqueness of Gauss curvature-based signatures of individual large earthquakes appears to hold with the refined epoch definitions. This refinement may add a valuable means to an existing research branch regarding the precursory signals of large earthquakes^4–6^. While being unique from other earthquakes’ signatures, there appears to exist interesting temporal variations of the Gauss curvature-based signatures as shown in Fig. 5C as getting close to the onset of a large earthquake. It is premature to draw any conclusion from this variation of the Gauss curvatures, but in the future extension the variations may be used for sequence-oriented ML methods^35,36^. For interested researchers, data sets of the refined epochs with one-day interval are shared on^28^.

Next, since the extreme earthquakes are rare, the data-driven prediction model most likely suffers from the overfitting problem. A successful prediction model may not generalize to other future earthquakes. But this issue may be tackled by the separation of two tasks—classification of large earthquakes and improvement of data-driven prediction models. With new data accumulate, the unique Gauss curvature-based signatures of individual large earthquakes can help reveal the difference or similarity of large earthquakes by using unsupervised ML techniques. Since an earthquake prediction model is customized for individual event, the separate classification task may inform us which prediction model should be used. Thereby, a prediction model can continue improving as being specialized for a class of large earthquakes, and there can be many different customized prediction models, in lieu of a single model. Moreover, the separate tasks of classification and model improvement can be performed by a high-level ML method such as a reinforcement learning^37^, which may facilitate ML-driven autonomous evolution.

The ML-identified best-so-far prediction model is not the final version but an interim candidate, being subject to substantial improvement and evolution. Since it is purely data-driven, the improvement of the earthquake data sets^22,24,38^ will positively influence the prediction accuracy. As long as the reliability and precision of the relevant data is ensured, further inclusion of more physics (e.g., thermal instability^1^, pore pressure^39^, fluid injection^2^) into the present framework would lead to a positive improvement, which will be straightforward in view of clear interpretability and extensibility of the present framework. There is ample room for further sophistication. It would be beneficial to consider more flexible, versatile bases^40^ for LFs, an extensive library of possible mathematical expressions^41^, powerful symbolic regression methods^42^, or stochastic optimizer^43^. Consistent evolution or automated optimization of many hyper-parameters of the framework may be done by inheriting the reinforcement learning paradigm^37^. This study may spark new research directions for seismogenesis. The smooth surface-like convolved information may catalyze the image-based deep learning methods. Gauss curvature-based signatures of large earthquakes may be clustered by unsupervised ML methods for better prediction models tailored for each category. Unique classifications of individual large earthquakes may advance the existing earthquake forecasting methods^5–8,10,12–15,31^. In light of the multifaceted nature of earthquake phenomena, enabling imminent individual earthquake predictions will require comprehensible collaborations^44^, and the outcome of this study will promote such a broad endeavor.

In essence, the primary novelty of this paper lies in the new feature generation processes, which first transform raw earthquake catalog data into spatio-temporal convolved information, then transform the convolved information into a number of pseudo physics-based features, and further transform the pseudo physics into smooth surface-like features using Gaussian curvatures. All these new features are then utilized by a transparent ML method (here, Bayesian evolutionary algorithm) to help identify unique signatures and to unravel hidden rules for prediction of large individual earthquake’s magnitude and location. Thereby, this paper’s outcome adds a new data- and ML-oriented dimension to the existing research paradigm for seismogenesis as well as natural hazards science and engineering.

## Methods

### Earthquake data homogenization within one-month epoch

Within one epoch (1 month), the spatial coordinates of every earthquake hypocenter available on the catalog are checked to determine which reference volume (i.e., a discretized volume in the lithosphere) the earthquake event belongs to. In particular, this study assumes that the hypocenter’s spatial coordinates $(\lambda , \phi , h)$ determine the associated reference volume. If an earthquake’s hypocenter resides in the *j*th reference volume’s domain ($\lambda _j \pm 0.1^\circ , \phi _j \pm 0.1^\circ , h_j \pm 5\, \text {km}$), it belongs to the reference volume. To some extent, this process homogenizes all the earthquake events within the reference volume in one epoch (1 month). All the impacts (e.g., the pseudo energy, power, vorticity, Laplacian, etc.) of the earthquakes within one reference volume in one epoch are homogenized and represented by the quantities at the center of the reference volume.

### Generation of new features using spatio-temporal information convolution

The raw data of earthquake hypocenters are assumed to be distributed over the lithosphere domain and thus constitute a sort of irregular 3D point cloud (Fig. 1A,B). This point cloud of hypocenters is used for all the following new features using the spatial and temporal convolutions. The observed earthquake hypocenter data sets adopted herein^27^ are processed into a text-based matrix form of $\{\lambda , \phi , -h, M \}_{i}^{(t)}$, $i=1,\ldots , n^{(t)}$ and $t=1,\ldots ,n_{ep}$ where $n^{(t)}$ means the number of total hypocenters recorded during one epoch (one month) in $[(t-1), t]$ and $n_{ep}$ means the number of total epochs (Table S1 summarizes the processed data from 1980 through 2019). One epoch is assumed to be one month, which may be adjustable for a specific scientific reason. The coordinates $\{\lambda ,\phi \}$ in [deg] stand for the longitude and latitude, respectively. The ground-normal *h* is in [km], being positive above the ground datum. The magnitude $M \in [0,10)$ means the observed moment magnitude. To facilitate the spatial convolution, the geodetic coordinates $\{\lambda , \phi , -h, M \}_{i}^{(t)}$ are transformed into the earth-centered rectilinear coordinate $\{x, y, z, M \}_{i}^{(t)}$ (see^29^). A point-wise information index (II) is denoted as “local” II, $II_{local}\in \mathbb {R}[0, 1]$ and calculated as ${II_{local}}^{(t)}(\mathbf{x} _i^{(t)}) = M_{i}^{(t)}/10$ where $\mathbf{x} _i^{(t)} = (x, y, z)_i^{(t)}$. The local II maps real earthquake magnitudes to the range of [0,1). Figure S1 shows the calculated point-wise information index during the periods between epoch 10465 and epoch 10476 (i.e. from October 2018 to September 2019;^27^). For comparison, the raw recorded magnitudes of relatively quiet epoch (10470) and active epoch (10474) are compared in Fig. 1A,B. Fault zones are inherently multiscale^2^ with a core being surrounded by the damaged zone of which macro-fractures decay with distance from the core^45^. Thus, an individual earthquake’s impact may not be described by a point-wise index, requiring a comprehensive means to capture a spatial impact on the surrounding. Complex spatial influences of many earthquakes are accounted for by the spatial convolution presented herein. If convolution is done over a spatial domain, ML can better understand the interaction of spatially distributed information and hidden patterns while applied to the temporal domain, the interactions between past and present information may be elucidated. This study seeks to spatially integrate the local II over the 3D point cloud, i.e., myriad earthquake events in the lithosphere. The key difference from the deep learning is that this study “externalizes” the multi-layered convolutions by conducting multiple convolutions at the information level, not in the opaque network layers. Rather than a uniform integration, we adopt a weighted integration using the Gaussian weight function (denoted $\omega$) to realize the proximity-proportionate importance of information. This process generates the “convolved spatial II;; denoted as $\overline{II}_S^{(t)}$. Figure S2C illustrates the derivation of the convolved spatial II. The physical meaning of the convolved spatial II is that $\overline{II}_S^{(t)}(\varvec{\xi }_{j}; L_k)$ quantifies how much the *j*th reference volume experiences earthquakes during one epoch (*t*) while the closer events the higher impact on the volume. The “reference volume” is defined as a discretized volume in the lithosphere with fixed spatial coordinate which is needed for spatial and temporal convolution (see details in “Methods” section and^29^). This study’s reference volume has dimensions of (0.1 deg, 0.1 deg, 5 km) due to the limit of computational resources. For a higher resolution, a finer reference volume may be adopted. If the earthquakes during the epoch took place nearby (i.e. within or close to the $L_k$) the failure directly affects the *j*th reference volume whereas earthquakes occurred at distance (i.e. much larger than $L_k$), the reduced impact is recorded in the *j*th reference volume via the $\overline{II}_S^{(t)}(\varvec{\xi }_{j}; L_k)$. This is a time-dependent quantity and thus defined at an epoch (*t*) and calculated as

9 $$\begin{aligned} \overline{II}_S^{(t)}(\varvec{\xi }_{j}; L_k) = \int _{\text {V}} { \omega (\varvec{\xi }_j, \mathbf{x} _{i}^{(t)}; L_k) II_{local}^{(t)}(\mathbf{x} _{i}^{(t)}) d\mathbf{x} } \end{aligned}$$

where $\omega (\varvec{\xi }_j, \mathbf{x} _{i}^{(t)}; L_k) = (L_k(2\pi )^{1/2})^{-N}\exp \left( -\frac{|\mathbf{x} _{i}^{(t)} - \varvec{\xi }_j|^2}{2L_k^2}\right) = \mathcal {N}(\mathbf{x} _{(i)}^{(t)}, L_k^2)$; $\varvec{\xi }_{j}$ stands for the position vector of the center of *j*th reference volume and $\forall \mathbf{x} _{i}^{(t)} \in \text {V}$, and V means the entire lithosphere domain under consideration. In this research, the 3D lithosphere domain is discretized into reference volumes—a fixed Eulerian 3D grid system. Thus, Eq. (9) is calculated by the discrete summation in lieu of continuous integration, of which details are presented in^29^. In Eq. (9), $L_k \in \mathbb {R}^{+}$, $k = 1,\ldots , n_L$ stands for the radius of influence range. With a larger value of $L_k$, the earthquake events across a broad space can be incorporated at the expense of over-smoothing effect; with a smaller $L_k$, higher priority on the adjacent earthquakes to the current reference volume at the expense of local spikes or over-fitting effect. For the weighting function, there is no restriction to the use of other weightings. The dimension parameter $N=3$ is used for the spatial convolution over the 3D point cloud whereas $N=1$ is used for the temporal convolution over time which shall be explained later. Fig. S2 shows three cases of the convolved spatial II with different influence ranges. All three cases used the procedures given in Eq. (9). Still, with a larger $L>50$ km such as Fig. S2C, the over-smoothing effect is notable. In heterogeneous materials or composite structures, this spatial convolved II may help ML understand internal complexity as scientists do^32–34,46,]^.

A reference volume in the lithosphere experiences incessantly many events over time. By extending convolution to the time domain, we can incorporate such transient information about how one reference volume has been being affected by past earthquakes. Performing convolution over time creates “convolved spatio-temporal II” (denoted as $\overline{II}_{ST}^{(t)}$). This convolved spatio-temporal II accounts for all the past earthquakes up to the present epoch *t*. Figure 1D illustrates the calculation procedure of the spatio-temporal II. The one-dimensional ($N=1$) Gaussian weighting is used, being centered at the present time *t*. Being not certain about the optimal temporal influence ranges, here we allow in total $n_T$ temporal influence ranges, denoted by $T_l, l=1,\ldots ,n_T$. For a temporal influence range $T_l$, we have

10 $$\begin{aligned} \overline{II}_{ST}^{(t)}(\varvec{\xi }_{j}; L_k, T_l) = \int { \omega (\tau ; T_l) \overline{II}_S^{(t_{past})}(\varvec{\xi }_{j}; L_k) dt_{past}} \end{aligned}$$

where $\omega (\tau ; T_l) = (T_l(2\pi )^{1/2})^{-1}\exp \left( -\frac{\tau ^2}{2T_l^2}\right) = \mathcal {N}(t, T_l^2)$; $\tau = |t-t_{past}|, t \ge t_{past}$, meaning the time gap between the current and the past time, all given in [epoch]. This convolved spatio-temporal II is calculated at the *j*th reference volume center, $\varvec{\xi }_{j}$. As the 3D lithosphere domain is discretized into a fixed Eulerian grid system of reference volumes, the continuous time space is discretized into non-overlapping epochs (one month each). Like the spatial convolved information Eq. (9), the spatio-temporal convolved information Eq. (10) is calculated by the discrete summation, of which details are presented in^29^.

As expected, the convolved spatio-temporal II appears to successfully quantify earthquake events (Fig. S5) and effectively distinguish the low and high seismic activities. The required numerical normalization and proofs of upper and lower bounds of the convolved IIs are provided in^29^.

### Pseudo released energy in terms of the convolved information

Earthquakes leave behind a footprint on energy^2,45^. The pseudo released energy (denoted as $E_r^{(t)}(\varvec{\xi }_{j}) \in \mathbb {R}^{+}$) of the *j*th reference volume at current time *t* may be represented in terms of the convolved spatio-temporal IIs. It should be noted that this paper does not adopt the well-known laws since the present goal is to pursue purely data-driven learning. Owing to the accumulated influences of adjacent earthquakes over time, it is plausible to consider that the pseudo released energy at a reference volume is increasing. Thus, the simple exponential form is preferred for the unknown expression of the pseudo released energy ($E_r$) in terms of the convolved spatio-temporal IIs ($\overline{II}_{ST}$). To identify the hidden expression of $E_r(\overline{II}_{ST})$, this paper leverages the Bayesian evolutionary algorithm in which an advanced evolutionary algorithm searches the vast space of parameters of the expression while the Bayesian update enables the probabilistic distributions of parameters to evolve as training proceeds with new data (see section “Combination of the Bayesian update and evolution algorithm” in^29^). Amongst many possible combination operations (e.g., $+$ or $\times$), the additive operation is found to be favorable. The best-so-far expression of the pseudo released energy identified by the Bayesian evolutionary algorithm is given by

11 $$\begin{aligned} E_r^{*(t)}(\varvec{\xi }_{j}) = \text {max}\left[ \sum _{k=1}^{n_L=2} \sum _{l=1}^{n_T=2} \mathcal {L}^{(k,l)}(\overline{II}_{ST}^{(t)}(\varvec{\xi }_{j}; L_k, T_l) ; ~\varvec{\uptheta }^{(k,l)}), 0.0\right] \end{aligned}$$

where $\varvec{\uptheta }^{(k,l)}$ is the best-so-far free parameters of the associated link function $\mathcal {L}^{(k,l)}$. Role of the link function $\mathcal {L}^{(k,l)}$ is to transform the convolved spatio-temporal information index $\overline{II}_{ST}^{(t)}(\varvec{\xi }_{j}; L_k, T_l)$ into a smooth, nonlinear output—here the pseudo released energy. In spirit, Eq. (11) resembles multiple layers of deep neural network as a nonlinear transformation route. Example plots using the exponential link functions (LFs) with the additive combination are shown in Fig. S7. The flexibility and expressibility of the LFs are explained in “Methods” and^29^. And the best combination of the spatial and temporal influence ranges is identified as $L_k=(10, 25)$ [km] and $T_l=(3, 6)$ [epoch = month]. This combination of short- and long-range influence ranges appears to outperform the other rules with a single *L* or *T* or many *L*’s and *T*’s. As shown in Fig. S8, the relative contribution of different influence ranges appears complicated but interpretable. In the higher II ranges ($\overline{II}_{ST}>$ 0.8), the spatio-temporal II with $(L_1, T_2)$ = (10 km, 6 epochs) and $(L_2, T_1)$ = (25 km, 3 epochs) to the pseudo released energy are significant (see Figs. S8B-C). In contrast, the contribution of II with $(L_1, T_1)$ = (10 km, 3 epochs) are uniform regardless of $\overline{II}_{ST}$ and thus important in the low and mid ranges of II ($\overline{II}_{ST}<$ 0.8; Fig. S8A). Although this identified rule of the pseudo released energy may not be close to the “exact” one, the clear interpretability of the pseudo released energy’s expression is still meaningful, conveying physically sound implications. For instance, Fig. S8A implies that nearly all the earthquakes in close distance and recent time retain their influence. Contrarily, Figs. S8B-C imply that only larger earthquakes ($\overline{II}_{ST}>$0.8) retain influence because they are far away or old enough to allow post-earthquake curing.

### Pseudo power, vorticity and Laplacian of the pseudo released energy

Other important physics quantities would be the spatial gradients of the pseudo released energy over the lithosphere and “power.” The time derivative of energy is physically related to the power. The calculation procedure of the time derivatives of the energy-related terms is presented in^29^. Figs. S9 and S10 present example plots of the spatial gradients and time derivative of the pseudo released energy at depth 2.5 km and 12.5 km, respectively. These plots are with respect to the earth-centered coordinate system before transformation to geodetic coordinates. The spatial gradient with respect to the geocentric coordinate system may convey weak physical and geometrical information in view of the curved structure of the earth lithosphere. Thus, it is meaningful to transform the geocentric gradient (denoted as $\nabla {E}_{r}^{(t)}$) to the geodetic gradient (denoted as $\nabla _{g} {E}_{r}^{(t)}$), i.e., the spatial gradient with respect to the geodetic coordinate system (${\lambda , \phi , h}$). This can be done by the Jacobian **J** (details are in^29^): $\nabla _{g} E_r^{(t)}(\varvec{\xi }_j) = \mathbf{J} \nabla E_r^{(t)}(\varvec{\xi }_j)$. Fig. S11 shows example plots of the gradient field vector at depth *z* = 12.5 km with respect to the geodetic coordinate system. By observing the transient change of the spatial gradient of the pseudo released energy, this study derives the pseudo “vorticity” $\varvec{\omega } = (\omega _\lambda , \omega _\phi , \omega _h)$ as

12 $$\begin{aligned}&\varvec{\omega } := \nabla _g \times \left( \nabla _g{ \frac{\partial E_r^{(t)}(\varvec{\xi }_{j})}{\partial {t}}} \right) \end{aligned}$$

13 $$\begin{aligned}&\quad = \left( \frac{\partial }{\partial \phi }\frac{\partial {E_r^{'}}}{\partial {h}} - \frac{\partial }{\partial {h}}\frac{\partial {E_r^{'}}}{\partial \phi }, \, \frac{\partial }{\partial {h}}\frac{\partial {E_r^{'}}}{\partial \lambda } - \frac{\partial }{\partial \lambda }\frac{\partial {E_r^{'}}}{\partial {h}}, \, \frac{\partial }{\partial \lambda }\frac{\partial {E_r^{'}}}{\partial \phi } - \frac{\partial }{\partial \phi }\frac{\partial {E_r^{'}}}{\partial \lambda } \right) \end{aligned}$$

In Eq. (12), $\nabla _g=(\partial /\partial \lambda ; \partial /\partial \phi , \partial /\partial {h})$;“$\times$” is the curl operator; $E_r'=\frac{\partial E_r^{(t)}(\varvec{\xi }_{j})}{\partial {t}}$. Fig. S12 presents example plots of the calculated vorticity vector. The vorticity of the pseudo released energy flow is considered as another physics quantity since the vorticity may hint at the temporal rotation of the strain energy field which may play an important role in rupture initiation. There is no direct definition of the velocity field needed for vorticity calculation, and thus the spatial gradient of the time derivative of the pseudo released energy ($\nabla _g{\frac{\partial E_r^{(t)}(\varvec{\xi }_{j})}{\partial {t}}}$) is regarded as a “pseudo velocity” in Eq. (12). Physically, this pseudo velocity field may describe the spatial distribution of how the pseudo released energy is changing over time. Although the time increment is large (here, one month) compared to the mathematical derivative, the slow motion of the earth plate (e.g., 8–10 cm/year^21^) may justify the use of such a large time interval for the pseudo velocity.

This study found that the higher-order gradient of the pseudo released energy also helps improve the prediction accuracy. In fact, such an important role of higher-order gradients can be easily found in many physics phenomena. For instance, in the heat transfer, the Laplacian of the externally observed temperature (*T*) is directly related to the temporal evolution of the internal heat energy, i.e., $\partial {T}/\partial {t}=\kappa \nabla ^2 T$ where $\kappa$ is the thermal diffusivity. This study calculates the “pseudo Laplacian” as

14 $$\begin{aligned} \nabla _g^2 E_r^{(t)}(\varvec{\xi }_{j}) = \frac{\partial ^2{E_r^{'}}}{\partial {\lambda ^2}}+ \frac{\partial ^2{E_r^{'}}}{\partial {\phi ^2}} + \frac{\partial ^2{E_r^{'}}}{\partial {h^2}} \end{aligned}$$

In addition to the resultant Laplacian in Eq. (14), this study compared individual term’s impact on prediction performance, i.e., prediction with $\frac{\partial ^2{E_r^{'}}}{\partial {\phi ^2}}, \frac{\partial ^2{E_r^{'}}}{\partial {\lambda ^2}}$, or $\frac{\partial ^2{E_r^{'}}}{\partial {h^2}}$. The comparative study found that the inclusion of the separate term of $\frac{\partial ^2{E_r^{'}}}{\partial {\lambda ^2}}$ in the prediction model showed a better accuracy.

### Calculation of the Gauss curvatures of the pseudo physics quantities

By regarding the distribution of a physics quantity at a certain depth as a smooth surface, this study can calculate the Gauss curvature at the maximum earthquake events. At a fixed depth *h*, let *u* stand for $\lambda$ and *v* for $\phi$. Let *Z*(*u*, *v*, *h*) stand for a physics quantity calculated at the depth *h* and the horizontal coordinates (*u*, *v*). *Z* can be any pseudo physics quantities such as the pseudo released energy, power, vorticity, and the Laplacian terms. Then, we can conceive a smooth surface $\varvec{X}(u,v,Z)$ at depth *h*. According to geometry, we can call $\varvec{X}$ as a map $\varvec{X}:\Omega \rightarrow \mathbb {E}^3$ where $\Omega$ is some open subset of plane $(u,v)\in \mathbb {R}^2$ and $\mathbb {E}^3$ is the Euclidean space where the standard inner product holds. And we assume $\varvec{X}$ to be $C^3$-continuous, i.e., all the partial derivatives up to the 3rd order exist and are continuous. This may be assured by the smoothness of all the pseudo physics quantities endowed by the spatio-temporal convolution process, and a rigorous mathematical proof is deferred to future extension. It is well known [e.g.,^47^] that the (total) Gauss curvature *K* is defined with the principal curvatures $\kappa _1$ and $\kappa _2$ as $K:=\kappa _1 \kappa _2$ of which detailed calculation procedures are presented in^29^. Using these Gauss curvature-based coordinates, the unique signature of individual large earthquake can be found by an exhaustive searching algorithm in Table 3.

**Table 3:** Algorithm—Searching the closest large earthquake using Gauss curvature coordinates.

**[Step-I] Calculate Gauss Curvature Coordinates**	
**Loop** *i* over all target epochs	
**Loop** *j* over all reference volumes	
Write the calculated principal Gauss curvature coordinates	
$$\mathbb {K}_j^{(i)} = [(\kappa _1, \kappa _2)_E, (\kappa _1, \kappa _2)_P, (\kappa _1, \kappa _2)_L, (\kappa _1, \kappa _2)_V]_j^{(i)}$$	
**End Loop** *i* and *j*	

**[Step-II] Compare Distances from the Reference Earthquakes**	
Get ready $\overline{\mathbb {K}}^{(k)}$	
where $k\in$ the set of epoch numbers of known large earthquakes (i.e. 8 events in Table 1)	
**Loop** *i* over all target epochs	
Calculate the mean absolute distance via L1 norm over all reference volumes	
$$k_{min}^{(i)} := \text {argmin}_{k \, \text {in} \forall j} ||\mathbb {K}_j^{(i)} - \overline{\mathbb {K}}^{(k)}||_1/8$$	
Store the found L1 norm and $k_{min}^{(i)}$ for each target epoch	
**End Loop** *i*	

### Reference volumes in the Earth lithosphere domain

This study defines reference volumes of the given domain in the Earth lithosphere. To generate 4D convolved spatio-temporal information index (II) by performing spatial and temporal convolutions, it is efficient to define a fixed location in the space and time, which is the central reason for adopting the Eulerian 3D grid system of reference volumes. Admittedly the Earth lithosphere is not a simple spherical structure, and the earthquake hypocenters are often recorded on longitude, latitude, and depth, $(\lambda ,\phi ,h)_i^{(t)}, i=1,\ldots , n^{(t)}$. Therefore, this study processes raw data to the earth-centered 3D coordinates, $(x,y,z)_i^{(t)}, i=1,\ldots , n^{(t)}$.

1. Transform Raw Hypocenter Data to Geocentric Coordinates: At current epoch (*t*), the first step is to read each hypocenter’s raw coordinate $(\lambda ,\phi ,-h)_i^{(t)}$ where the longitude $x\lambda \in [-180, 180]$ is in [deg], the latitude $\phi \in [-90, 90]$ in [deg], and the depth *h* in [km]. Here, *h* means the ellipsoidal height along its normal, being positive outward normal to the reference ellipsoid. Note the earthquake catalog data use the reversed sign convention of *h*. Transform them to the earth-centered 3D spatial coordinate $(x, y, z)_i^{(t)}$ (i.e. geocentric rectangular coordinates) as described in^48^
  15 $$\begin{aligned}&x = \left( \frac{a^2}{\sqrt{a^2 \text {cos}^2 \phi + b^2 \text {sin}^2 \phi }} + h \right) \text {cos}\phi \; \text {cos}\lambda \end{aligned}$$
  16 $$\begin{aligned}&y = \left( \frac{a^2}{\sqrt{a^2 \text {cos}^2 \phi + b^2 \text {sin}^2 \lambda }} + h \right) \text {cos}\phi \; \text {sin}\lambda \end{aligned}$$
  17 $$\begin{aligned}&z = \left( \frac{b^2}{\sqrt{a^2 \text {cos}^2 \phi + b^2 \text {sin}^2 \lambda }} + h \right) \text {sin}\phi \end{aligned}$$
  where $a=6378.1370$ km and $b=6356.7523$ km according to the 1984 World Geodetic System (WGS 84) revision.
2. Reference Volumes: Given the ranges of longitudes, latitudes, and depths, this study defines uniformly distributed grid system, and each cell is denoted as a reference volume. The total number of reference volumes $n_{rv}$ is simply calculated by $n_{rv} = n_{\lambda } \times n_{\phi } \times n_{h}$ where $n_{\lambda } = |\lambda _{max} - \lambda _{min}|/\Delta {\lambda }$, $n_{\phi } = |\phi _{max} - \phi _{min}|/\Delta {\phi }$, and $n_{h} = |h_{max} - h_{min}|/\Delta {h}$. Here $(\Delta {\lambda }, \Delta {\phi }, \Delta {h})$ are user-defined increments of longitude, latitude, and depth, respectively. The index of reference volume is ordered by $\lambda , \phi ,$ and *h*. Thus, the coordinates or the *j*th reference volume’s center, denoted as $\varvec{\xi }_j\in \mathbb {R}^3$, is represented by
  18 $$\begin{aligned} \varvec{\xi }_j = \left( \lambda _{min}+(j_{\lambda }+\frac{1}{2})\Delta {\lambda }, {\phi }_{min}+(j_{\phi }+\frac{1}{2})\Delta {\phi }, -{h}_{min}-(j_h+\frac{1}{2})\Delta {h} \right) , \end{aligned}$$
  where $j=j_{\lambda } + j_{\phi }\times n_{\lambda } + j_h \times (n_{\lambda } \times n_{\phi }), j_{\lambda } \in \mathbb {Z}[0,n_{\lambda }-1], j_{\phi } \in \mathbb {Z}[0,n_{\phi }-1],$ and $j_h \in \mathbb {Z}[0,n_h-1].$ After calculating the center coordinates in ([deg], [deg], [km]), we can easily transform them to the geocentric rectangular coordinates using the same formulae in Eqs. (15–17). Whenever using Eq. (17), the outward normal is used for the positive sign of the depth. Another important quantity about the reference volume is the actual volume of individual reference volume element. In view of the curved ellipsoidal lithosphere, the volume of the *j*th reference volume element $V_j$ [$\text {km}^3$] is calculated by
  19 $$\begin{aligned} V_j = 4\pi \Vert \varvec{\xi }_j \Vert ^2_2 \Delta h \frac{\Delta \lambda }{360^\circ } \frac{\Delta \phi }{180^\circ } \end{aligned}$$

### Computational implementation of proposed algorithms

The spatio-temporal convolution of earthquake catalog data to generate the convolved II is computationally expensive. This study developed a parallelized Bayesian evolutionary algorithm framework with *C++* and *OpenMPI*. All other learning, evolutionary algorithm and Bayesian update scheme are implemented on the parallel program. The developed program is made available upon request to the author. Iowa State University’s high-performance computing facility, *Condo* cluster is used for this study.

### Flexible and transparent link functions

Placing top priority on the interpretability, this study proposes to adopt an expressive link function (LF) using transparent, flexible basis that can describe a mathematical expression between the convolved spatio-temporal II, $\overline{II}_{ST}$ and the hidden physical rules. LF is denoted as $\mathcal {L}(\overline{II}_{ST}; \varvec{\uptheta })$ where $\varvec{\uptheta }$ is a set of free parameters prescribing the LF. This study used an evolutionary algorithm coupled with the Bayesian update scheme to enable LF to continue to learn, train, and evolve. There is little restriction of choice of other forms of LFs. For balancing the efficiency and interpretability, one may choose the cubic regression spline (CRS)-based LF with high flexibility^40,49^ or two-parameter based exponential LF with its simplicity. First, the CRS-based LF has a general form as $\mathcal {L}^{(k,l)}(\overline{II}_{ST}^{(t)}(\varvec{\xi }_{j}; L_k, T_l) ; ~\varvec{\uptheta }^{(k,l)}) = \sum _{i=1}^p{a_{i}^{(k,l)} b_{i}^{(k,l)}(\overline{II}_{ST}^{(t)}(\varvec{\xi }_{j}; L_k, T_l))}$ where ${\varvec{\uptheta }}^{(k,l)} = \{\mathbf{a }, \mathbf{x }^{*}\}^{(k,l)}$ with $\mathbf{a} ^{(k,l)} = \{a_1,\ldots ,a_p\}^{(k,l)}$, the knots $\mathbf{x} ^{*(k,l)} = \{x_1^*,\ldots ,x_{(p-2)}^*\}^{(k,l)}$, and the cubic spline basis $b_{i}^{(k,l)}$ given in Eq. (33) in^29^. Next, the two-parameter exponential LF has a simpler form as $\mathcal {L}^{(k,l)}(\overline{II}_{ST}^{(t)}(\varvec{\xi }_{j}; L_k, T_l) ; ~\varvec{\uptheta }^{(k,l)}) = \text {exp}\left( a^{(k,l)}\overline{II}_{ST}^{(t)}(\varvec{\xi }_{j}; L_k, T_l)^{b^{(k,l)}}\right) - 1$ where $\varvec{\uptheta }^{(k,l)} = \{a^{(k,l)}, b^{(k,l)}; k=1,\ldots ,n_L, l=1,\ldots ,n_T\}$, and “-1” is to make the minimum of the LF near zero. It should be noted that the exponential LF is always non-zero, positive, and monotonically increasing while preserving the concave or convex shape (see Fig. S6 in^29^).

## Supplementary Information

Supplementary Information.

## Data Availability

The processed 40-years data sets consisting of the month-based epochs and the refined day-based epochs are shared on a cloud storage^28^. Other supplementary data and parallel programs supporting other findings of this paper will be available upon request to the author.
