# Supplementary material for: Gauss curvature-based unique signatures of individual large earthquakes and its implications for customized data-driven prediction
Source: Sci Rep. 2022 May 23;12:8669. doi: 10.1038/s41598-022-12575-w (PMC9127126; doi:10.1038/s41598-022-12575-w)
Supplement: Supplementary file 1 — Supplementary Information. [file 41598_2022_12575_MOESM1_ESM.pdf]

Gauss Curvature-Based Unique Signatures of Individual Large Earthquakes and Its Implications for Customized Data-Driven Prediction

In Ho Cho[1]

[1]**CCEE Department, Iowa State University, Ames, IA 50011, USA**

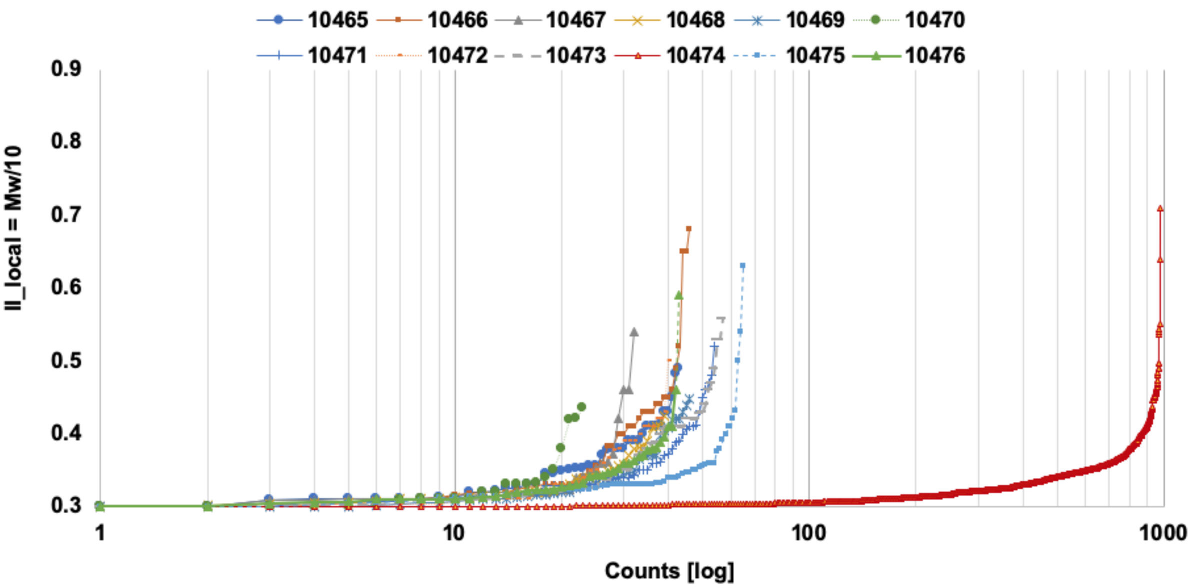

Fig. S1. Point-wise information index, II_{local}. Events above 0.3 (i.e. $M_w \geq 3.0$) are shown. Each line corresponds to the events during one-month period from October 2018 (epoch number 10465) to September 2019 (10476).

Rescaling of the convolved information index

The spatio-temporal convolved information index (II) may span substantially small values which may hamper the searching power of the adopted link function (LF). For instance, the cubic regression spline (CRS)-based LF takes a covariate ranging between [0,1], and thus if the covariate is very small the subsequent learning may not be efficient. Thus, it is helpful to rescale the convolved spatio-temporal II to [0,1]. One immediate normalization would be using the

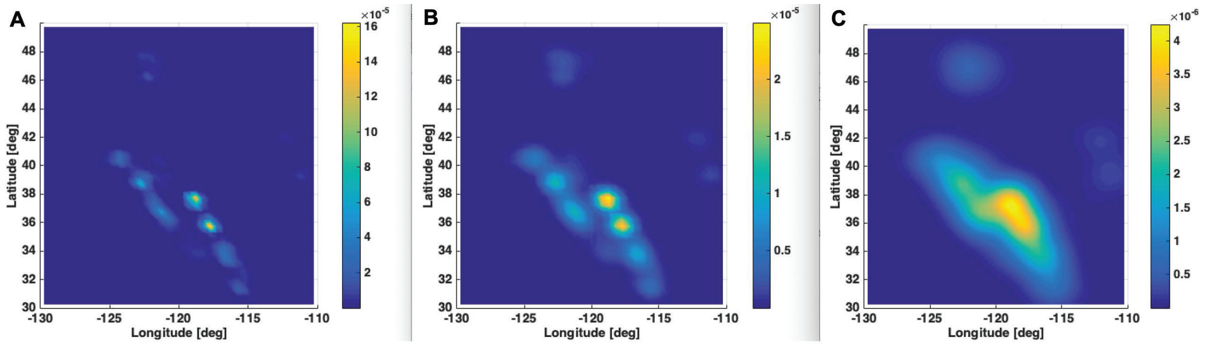

Fig. S2. Convolved Spatial II at the depth z = 12.5 km: **(A)** Calculated with $L = 25$ km. **(B)** With $L = 50$ km. **(C)** $L = 100$ km.

maximum range of the convolved spatio-temporal II during the current epoch (t) as

$$\overline{II}^{(t)}_{ST}(\boldsymbol{\xi}_j; L_k, T_l) = \frac{\overline{II}^{(t)}_{ST}(\boldsymbol{\xi}_j; L_k, T_l) - \text{min}_{\forall\, \boldsymbol{\xi}_j}[\overline{II}^{(t)}_{ST}(\boldsymbol{\xi}_j; L_k, T_l)]}{|\text{max}_{\forall\, \boldsymbol{\xi}_j}[\overline{II}^{(t)}_{ST}(\boldsymbol{\xi}_j; L_k, T_l)] - \text{min}_{\forall\, \boldsymbol{\xi}_j}[\overline{II}^{(t)}_{ST}(\boldsymbol{\xi}_j; L_k, T_l)]|} \tag{20}$$

However, this simple normalization may not guarantee consistency over different epochs since each epoch may have different ranges of the spatio-temporal IIs. Therefore, it is more robust to use the normalization with the upper bound (see a brief proof in the following section) of the spatio-temporal II as

$$\overline{II}^{(t)}_{ST}(\boldsymbol{\xi}_j; L_k, T_l) = \frac{\overline{II}^{(t)}_{ST}(\boldsymbol{\xi}_j; L_k, T_l)}{\widetilde{n}_{epoch}(T_l(2\pi)^{1/2})^{-1} \times \widetilde{n}^{(t)}(L_k(2\pi)^{1/2})^{-3}} \tag{21}$$

where it is reasonably assumed that $\widetilde{n}_{epoch} = 60$ epochs (i.e., 5 years); $\widetilde{n}^{(t)} = 200$ (i.e. at most 200 events larger than magnitude 3.0 per month) based on the past earthquakes in the

Table S1. Earthquake (EQ) catalog and the associated epochs [1 month = 1 epoch]

Year (begin)	Year (end)	Epoch (begin)	Epoch (end)	Total EQs
1980	1984	10000	10059	152,328
1985	1989	10060	10119	173,414
1990	1994	10120	10179	241,141
1995	1999	10180	10239	219,382
2000	2004	10240	10299	214,145
2005	2009	10300	10359	252,833
2010	2014	10360	10419	320,211
2015	2019 (up to Oct.)	10420	10477	321,736

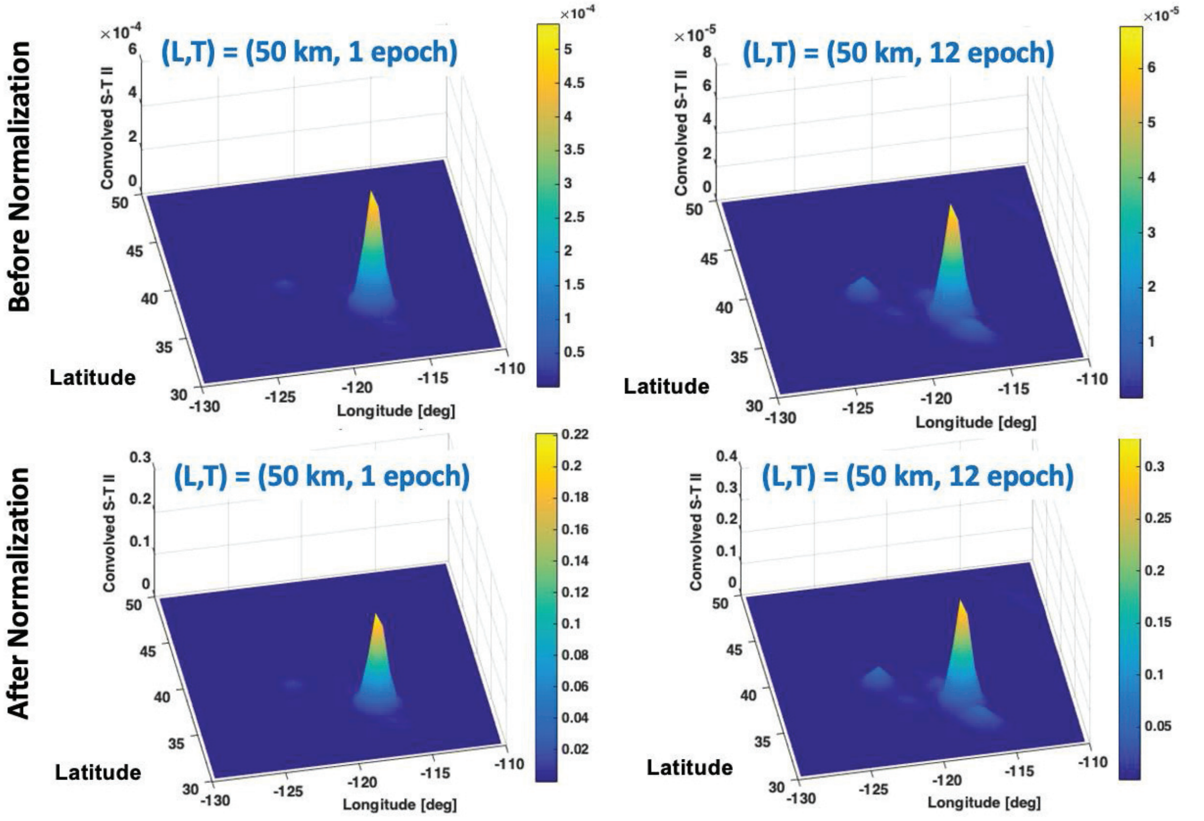

Fig. S3. Convolved spatio-temporal II after normalization with the physical upper bound. The same shape but dramatic increase in the scale of values from 10^{-5} to the desired range of [0,1].

catalog database (*41*). This assumption of constant $\widetilde{n}_{epoch}$ and $\widetilde{n}^{(t)}$ helps the spatio-temporal II be consistently normalized for a given pair of (L_k, T_l). As long as the consistency is being held and the resulting II is within [0,1], these values of $\widetilde{n}_{epoch}$ and $\widetilde{n}^{(t)}$ can be changed by researchers for the upper bound-based normalization. Fig. S3 presents the positive role of the upper bound-based normalization that boosts the convolved spatio-temporal II to the manageable range of [0,1]. To consider the upper bound of the spatio-temporal II, it is necessary to consider the upper bound of the convolved spatial II. In lieu of exploring all possible ranges, bound-aware searching helps fast searching, and thus the derivation of the upper bound is presented in the following section.

Upper bound of spatio-temporal information index

To consider the upper bound of the spatio-temporal II, it is necessary to consider the upper bound of the convolved spatial II.

$$\overline{II}_S^{(t)}(\boldsymbol{\xi}_j; L_k) \cong \sum_{i=1}^{n^{(t)}} \omega(\boldsymbol{\xi}_j, \mathbf{x}_i^{(t)}; L_k) II_{local}^{(t)}(\mathbf{x}_i^{(t)}) V(\mathbf{x}_i^{(t)}) \tag{22}$$

$$\leq \sum_{i=1}^{n^{(t)}} \omega(\boldsymbol{\xi}_j, \mathbf{x}_i^{(t)}; L_k) \times 1.0 \times 1.0 \tag{23}$$

$$\leq n^{(t)} \times \omega(\boldsymbol{\xi}_j = \mathbf{x}_i^{(t)}; L_k) = n^{(t)} \times (L_k(2\pi)^{1/2})^{-3} \tag{24}$$

Here, the inequality Eq. 23 assumes the extreme case when all events are maximum magnitude of 10 and thus $II_{local} = 10/10 = 1.0$. $V(\mathbf{x}_i^{(t)}) = 1.0$ is physically explained in the text around Eq. 29. The last inequality Eq. 24 assumes another extreme case when all events happen at the jth reference volume and all the Gaussian weights take the maximum value. Thus, the maximum physically conceivable upper bound of $\overline{II}_S^{(t)}(\boldsymbol{\xi}_j; L_k)$ is $n^{(t)}(L_k(2\pi)^{1/2})^{-3}$. Now we can consider the upper bound of the convolved spatio-temporal II.

$$\overline{II}_{ST}^{(t)}(\boldsymbol{\xi}_j; L_k, T_l) \cong \sum_{i=1}^{n_{epoch}} \omega(\tau_i; T_l) \overline{II}_S^{(t_i)}(\boldsymbol{\xi}_j; L_k) \Delta t_i \tag{25}$$

$$\leq \sum_{i=1}^{n_{epoch}} \omega(\tau_i; T_l) \left(n^{(t_i)} (L_k(2\pi)^{1/2})^{-3} \right) \Delta t_i \tag{26}$$

$$\leq n_{epoch} \times \omega(\tau_i = 0; T_l) \left(n^{(t_i)} (L_k(2\pi)^{1/2})^{-3} \right) \Delta t_i \tag{27}$$

$$\leq \left(n_{epoch} \times (T_l(2\pi)^{1/2})^{-1} \right) \left(n^{(t_i)} (L_k(2\pi)^{1/2})^{-3} \right) \Delta t_i \tag{28}$$

The inequality Eq. 27 assumes the extreme case that all past earthquake events took place current epoch and thus gives $\tau_i = 0$. As explained before, $\forall \Delta t_i = 1$ epoch which means 1 month in this study. Therefore, the physically conceivable upper bound of the convolved spatio-temporal II is $\left(n_{epoch}(T_l(2\pi)^{1/2})^{-1}\right) \left(n^{(t_i)}(L_k(2\pi)^{1/2})^{-3}\right)$.

Discretization of convolved information index

For the integration over a discrete 3D point cloud, with the uniformity assumption over a reference volume, the approximation is given by

$$\overline{II}_S^{(t)}(\boldsymbol{\xi}_j; L_k) \cong \sum_{i=1}^{n^{(t)}} \omega(\boldsymbol{\xi}_j, \mathbf{x}_i^{(t)}; L_k) II_{local}^{(t)}(\mathbf{x}_i^{(t)}) V(\mathbf{x}_i^{(t)}) \tag{29}$$

where $V(\mathbf{x}_i^{(t)})$ is the volume which contains the ith hypocenter in the lithosphere domain at time (t) and is assumed to be 1 km^3. Rationales behind this unit volume assignment to each hypocenter point's $V(\mathbf{x}_i^{(t)})$ are twofold. First, the spatial impact of each hypocenter is already considered by the II_{local}'s weighted spatial integration in Eqs. (9) and (29). Second, each epoch has new hypocenters emerging at the random locations and with a random total count. To retain the physical consistency of the II, i.e. the more earthquakes the more released strain energy, it is efficient to use the unit volume per hypocenter. Unlike $V(\mathbf{x}_i^{(t)})$, the jth the reference volume does have actual spatial volume (denoted as V_j) which is accurately calculated on the Earth ellipsoid reference (see the algorithm in corresponding section in (*37*)). For the discretization of the integration over the time space,

$$\overline{II}_{ST}^{(t)}(\boldsymbol{\xi}_j; L_k, T_l) \cong \sum_{i=1}^{n_{epoch}} \omega(\tau_i; T_l) \overline{II}_S^{(t_i)}(\boldsymbol{\xi}_j; L_k) \Delta t_i \tag{30}$$

where $\tau_i = |t - t_i|, t \geq t_i$ and Δt_i is the incremental time lapse over one epoch, which is assumed to be constant $\Delta t_i = 1$ epoch (i.e. one month). $T_l \in \mathbb{R}^+$, $l = 1, ..., n_T$. With a larger value of T_l, the earthquake events across a longer past period can be incorporated at the expense of over-smoothing effect; with a smaller T_l, a higher priority on the most recent earthquakes to the present time at the expense of local spikes or over-fitting effect. Choosing values and total counts of T_l is subject to learning and prediction accuracy. To some extent, this inclusion of temporal effects by ML shares the similar notions of the well-known long short-term memory (LSTM) and its variants that uses selective remembering and forgetting in the time axis.

Training with a frame of epochs

Fig. S4 explains the training with a frame of epochs. Departing from the first epoch, all the spatio-temporal convolved IIs within the frame will be used for training and then the identified rule is used to predict the earthquakes of the last epoch of the frame. Departing from the first epoch (marked by "*START_EPOCH_NUMBER*") with the length of "*NUMBER_TOTAL_EPOCH_DATA_SETS*", all the convolved spatio-temporal IIs within the frame of epochs are used for training and then the identified rule is used to predict the earthquakes of the last epoch of the frame (marked by dashed box). After training, the best-so-far rules are identified. For instance, when "*START_EPOCH_NUMBER* = 10462", and "*NUMBER_TOTAL_EPOCH_DATA_SETS* = 12", the training is conducted with $\forall II_{ST}^{(t)}, t = [10462, 10472]$ (in total 11 epochs) in order to identify the hidden rules that can best reproduce earthquakes happening at the last epoch 10473. These identified rules are stored as a prior generation. Between different frames, the prior best generation of the identified rules can be inherited via the combination of Bayesian update and evolutionary algorithm.

Flexible and expressive link functions

Placing top priority on the interpretability, this study proposes to adopt an expressive link function (LF) using transparent, flexible basis that can describe a mathematical expression between the convolved spatio-temporal II, $\overline{II}_{ST}$ and the hidden physical rules. LF is denoted as $\mathcal{L}(\overline{II}_{ST}; \theta)$ where θ is a set of free parameters prescribing the LF. This study used an evolutionary algorithm coupled with the Bayesian update scheme to enable LF to continue to learn, train, and evolve. There is little restriction of choice of other forms of LFs. For balancing the efficiency and interpretability, this study chose the cubic spline basis owing to its high smoothness and flexibility. The cubic spline curves consist of a few cubic polynomials connected at knots so that the curves are continuous up to the second derivatives (*40*). If practical cubic spline

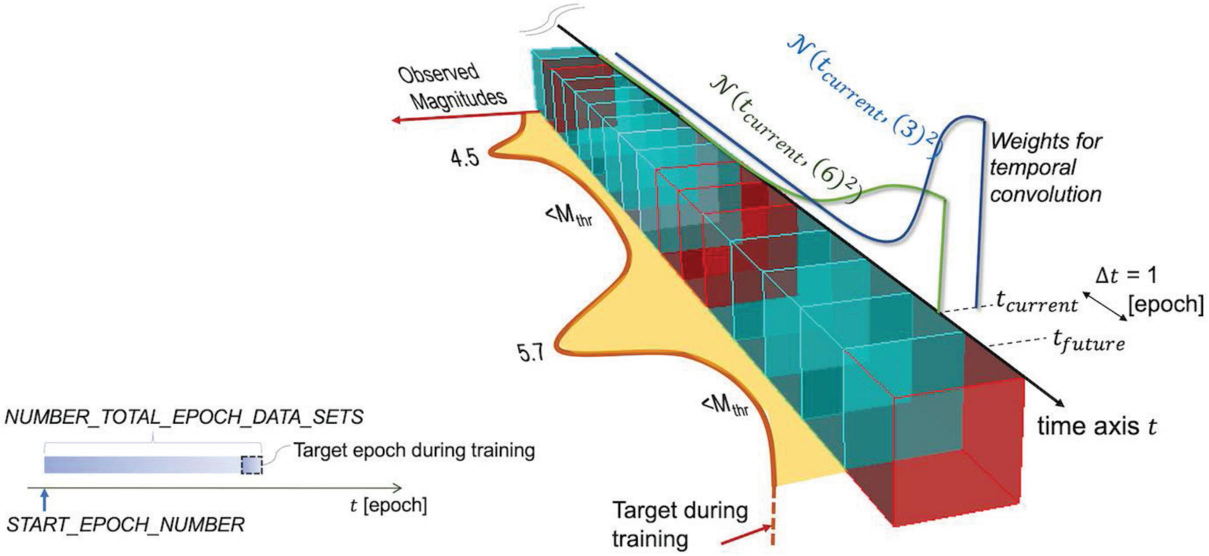

Fig. S4. Illustration of training with a frame of epochs. The last epoch of frame is used for target during training. Between different frames, the Bayesian inheritance of the prior-best rules may take place.

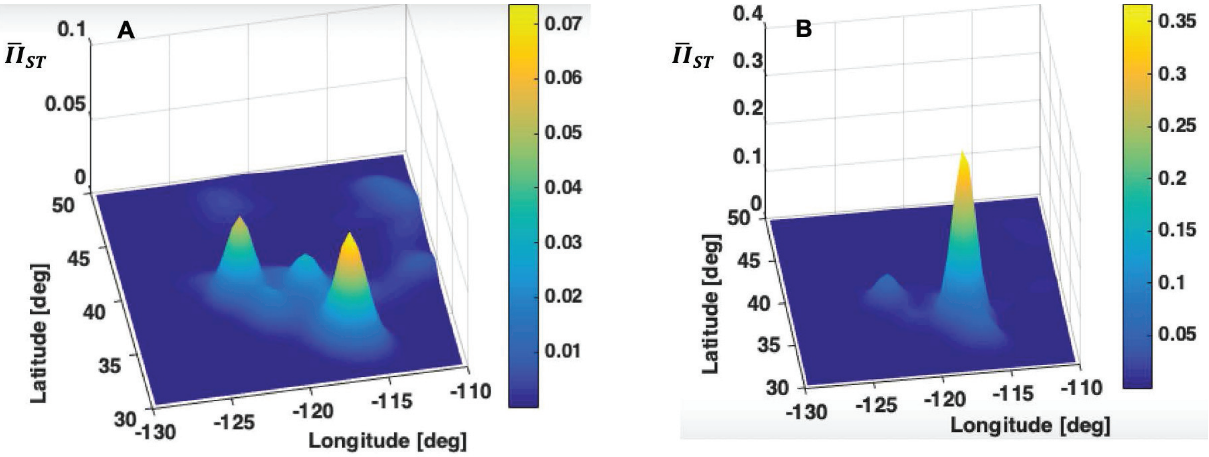

Fig. S5. Comparison of two convolved spatio-temporal IIs: (A) $\overline{II}_{ST}$ over 12 epochs from 10462 to 10472 showing relatively low seismic activity; **(B)** $\overline{II}_{ST}$ over 12 epochs from 10465 to 10475 showing relatively high seismic activity with peaks fivefold larger than **(A)**, mainly due to the notably active epoch 10474 (see Fig. S1 and Fig. 1). All $\overline{II}_{ST}$s are generated with (L, T) =(75 km, 12 epochs) and the plot depth is $z = 12.5$ km.

bases (*47*) (denoted as b_i) are adopted, LFs are given as

$$\mathcal{L}(\overline{II}_{ST};\ \mathbf{a}, \mathbf{x}^*) = \sum_i^p a_i b_i(\overline{II}_{ST}) \tag{31}$$

$$\textbf{CRS-Based LF: } \mathcal{L}^{(k,l)}(\overline{II}_{ST}^{(t)}(\boldsymbol{\xi}_j; L_k, T_l);\ \theta^{(k,l)}) = \sum_{i=1}^p a_i^{(k,l)} b_i^{(k,l)}(\overline{II}_{ST}^{(t)}(\boldsymbol{\xi}_j; L_k, T_l)); \tag{32}$$

where $b_1(x) = 1, b_2(x) = x$, and

$$b_{i+2}(x) = \frac{[(x_i^* - \frac{1}{2})^2 - \frac{1}{12}][(x - \frac{1}{2})^2 - \frac{1}{12}]}{4} - \frac{[(|x - x_i^*| - \frac{1}{2})^4 - \frac{1}{2}(|x - x_i^*| - \frac{1}{2})^2 + \frac{7}{240}]}{24}, \tag{33}$$

for $i = 1...p-2$. Here, x_i^* is i_{th} knot location. To fully describe one LF, we need to identify $p+(p-2)$ unknowns, i.e. $\mathbf{a} = \{a_1, ..., a_p\}$ and $\mathbf{x}^* = \{x_1^*, ..., x_{(p-2)}^*\}$. For brevity, we denote the total unknown parameters as $\theta = \{\mathbf{a}, \mathbf{x}^*\}$ hereafter. The adopted cubic spline bases can accommodate a variety of relation forms, ranging from a simple monotonic rule to a highly nonlinear rule. It should be noted that the adopted cubic spline basis is not for the direct regression, but for the transparent expressions of the final rule.

$$\textbf{Exponential LF: } \mathcal{L}^{(k,l)}(\overline{II}_{ST}^{(t)}(\boldsymbol{\xi}_j; L_k, T_l);\ \theta^{(k,l)}) = \exp\left(a^{(k,l)}\overline{II}_{ST}^{(t)}(\boldsymbol{\xi}_j; L_k, T_l)^{b^{(k,l)}}\right) - 1 \tag{34}$$

Time derivatives and spatial gradient of the pseudo released energy

The time derivative of energy is physically related to the power. For later use of power term in the prediction model it is helpful to prepare time derivatives of the energy-related terms. For the finite difference method, we adopt the backward difference scheme in view of our goal to predict the imminent earthquake in the next time epoch $(t+1)$, and thus the form is given as

$$\frac{\partial \overline{II}_{ST}^{(t)(k,l)}}{\partial t} = \frac{\overline{II}_{ST}^{(t)(k,l)} - \overline{II}_{ST}^{(t-1)(k,l)}}{\Delta t} + \mathcal{O}(\Delta t) \tag{35}$$

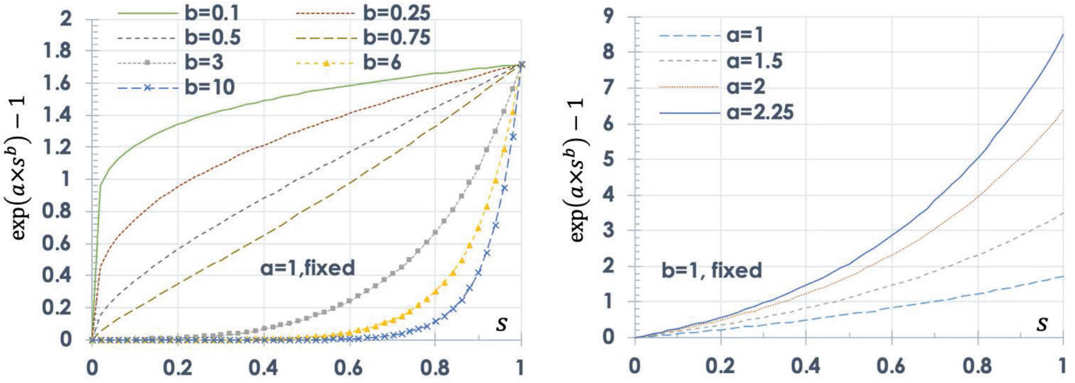

Fig. S6. Exponential LF: The two-parameter exponential LF is adjusted by a and b. While a controls the amplitude, b governs the shape.

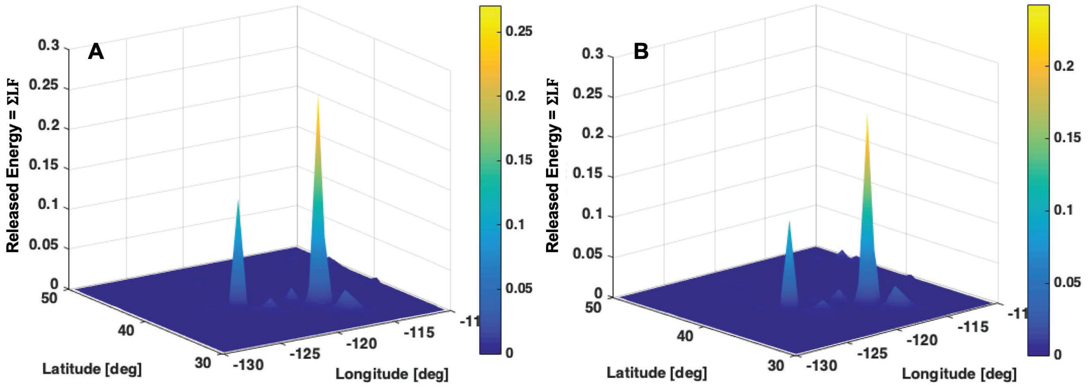

Fig. S7. Pseudo released energy by additive combination of exponential LFs: Example plots of the pseudo released energy calculated by the additive combination of four exponential LFs with (L, T), $L = (12.5, 25)$ km and $T = (6, 12)$ epochs **(A)** at the depth $z = 7.5$ km and **(B)** $z = 12.5$ km.

$\partial a^{(t)(k,l)}/\partial t$ and $\partial b^{(t)(k,l)}/\partial t$ are similarly calculated. It should be noted that the spatial gradient is with respect to the geocentric coordinate system which convey little physical and geometrical information of the earth lithosphere. Thus, it is meaningful to transform the geocentric gradient of a function f to the geodetic gradient (denoted as ∇_gf), i.e., the gradient with respect to the geodetic coordinate system λ, ϕ, h. This can be done by multiplying the Jacobian $\mathbf{J}$ as

$$\nabla_g \mathrm{f}(\boldsymbol{\xi}_j) = \mathbf{J}\nabla \mathrm{f}(\boldsymbol{\xi}_j), \tag{36}$$

where the Jacobian's entities $\{J_{(i,j)}\}$, $i, j = 1, 2, 3$ as given by

$$J_{(1,1)} = \frac{\partial x}{\partial \lambda} = -\left(DJ_1\right)\cos\phi\ \sin\lambda \tag{37}$$

$$J_{(2,1)} = \frac{\partial x}{\partial \phi} = \left(DJ_2\right)\cos\phi\ \cos\lambda - (DJ_1)\sin\phi\ \cos\lambda \tag{38}$$

$$J_{(3,1)} = \frac{\partial x}{\partial h} = \cos\phi\ \cos\lambda \tag{39}$$

$$J_{(1,2)} = \frac{\partial y}{\partial \lambda} = \left(DJ_1\right)\cos\phi\ \cos\lambda \tag{40}$$

$$J_{(2,2)} = \frac{\partial y}{\partial \phi} = \left(DJ_2\right)\cos\phi\ \sin\lambda - (DJ_1)\sin\phi\ \sin\lambda \tag{41}$$

$$J_{(3,2)} = \frac{\partial y}{\partial h} = \cos\phi\ \sin\lambda \tag{42}$$

$$J_{(1,3)} = \frac{\partial z}{\partial \lambda} = 0 \tag{43}$$

$$J_{(2,3)} = \frac{\partial z}{\partial \phi} = \left(DJ_4\right)\sin\phi + (DJ_3)\cos\phi \tag{44}$$

$$J_{(3,3)} = \frac{\partial z}{\partial h} = \sin\phi \tag{45}$$

$$DJ_1 = \frac{a^2}{(a^2\cos^2\phi + b^2\sin^2\phi)^{1/2}} + h \tag{46}$$

$$DJ_2 = \frac{a^2(a^2\cos\phi\ \sin\phi - b^2\sin\phi\ \cos\phi)}{(a^2\cos^2\phi + b^2\sin^2\phi)^{3/2}} \tag{47}$$

$$DJ_3 = \frac{b^2}{(a^2\cos^2\phi + b^2\sin^2\phi)^{1/2}} + h \tag{48}$$

$$DJ_4 = \frac{b^2(a^2\cos\phi\ \sin\phi - b^2\sin\phi\ \cos\phi)}{(a^2\cos^2\phi + b^2\sin^2\phi)^{3/2}} \tag{49}$$

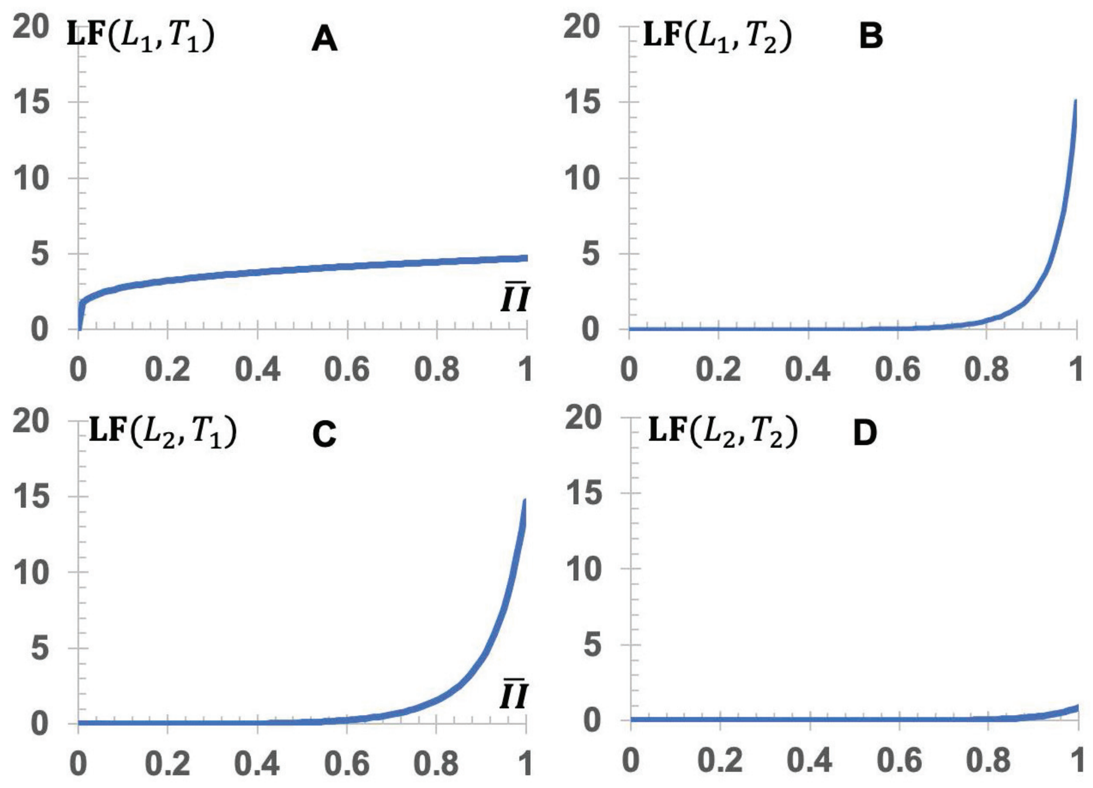

Fig. S8. Link function comparison of the identified best-so-far expression of the pseudo released energy. **(A)** Two-parameter exponential LF with (L_1, T_1) =(10 km, 3 epochs). **(B)** (L_1, T_1) =(10 km, 6 epochs). **(C)** (L_2, T_1) =(25 km, 3 epochs). **(D)** (L_2, T_2) =(25 km, 6 epochs).

Detailed calculation procedures of the Gauss curvatures

The total Gauss curvature K is defined with the principal curvatures κ_1 and κ_2 as

$$K := \kappa_1 \kappa_2 \tag{50}$$

$$\kappa_1 = H + C;\ \kappa_2 = H - C \tag{51}$$

where

$$H = \frac{GL - 2FM + EN}{2(EG - F^2)} \tag{52}$$

$$C = \sqrt{A^2 + B^2} \tag{53}$$

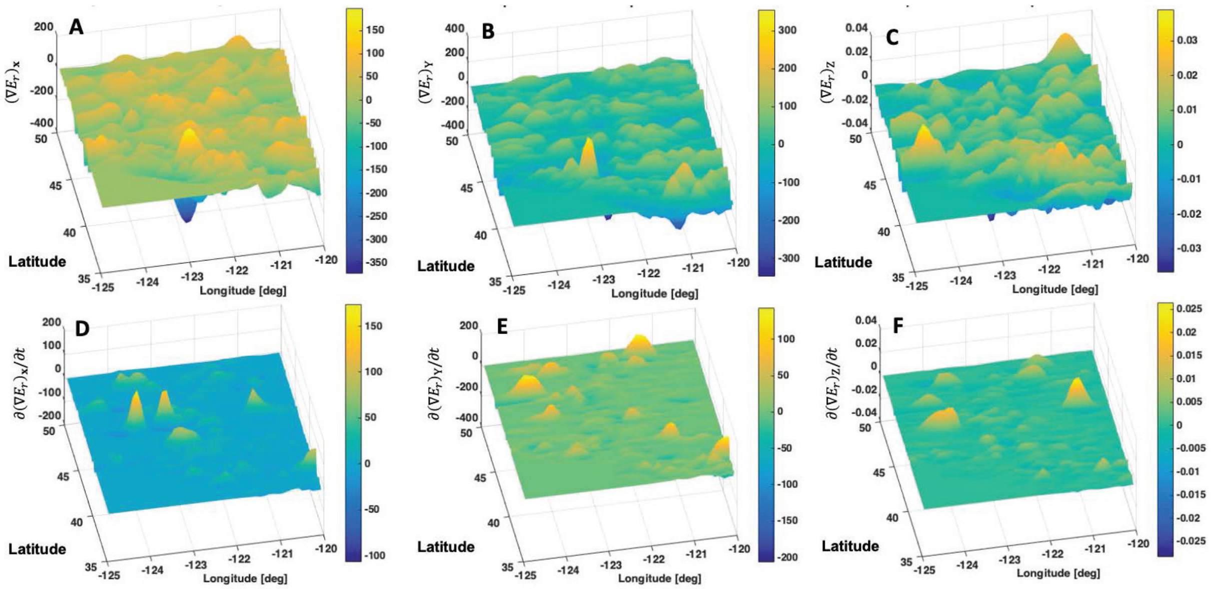

Fig. S9. Spatial gradients and time derivatives of the pseudo released energy at depth = 2.5 km generated from the past 10 years data (epochs from 10355 through 10473). (A-C) At depth = 2.5 km, three components of the spatial gradients of the pseudo released energy with respect to the earth-centered XYZ system. **(D-F)** Time derivative of the spatial gradients of the pseudo released energy with the time increment of 1 epoch for derivative calculation.

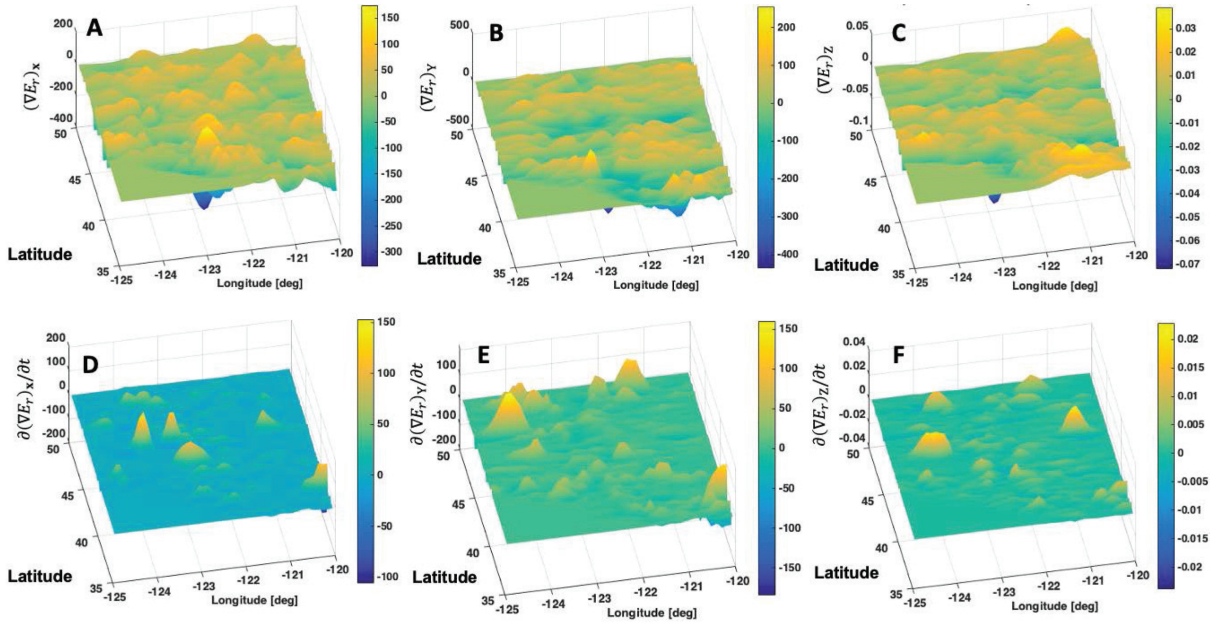

Fig. S10. Spatial gradients and time derivatives of the pseudo released energy at depth = 12.5 km generated from the past 10 years data (epochs from 10355 through 10473). (A-C) Three components of the spatial gradients of the pseudo released energy with respect to the earth-centered XYZ system. **(D-F)** Time derivative of the spatial gradients of the pseudo released energy with the time increment of 1 epoch for derivative calculation.

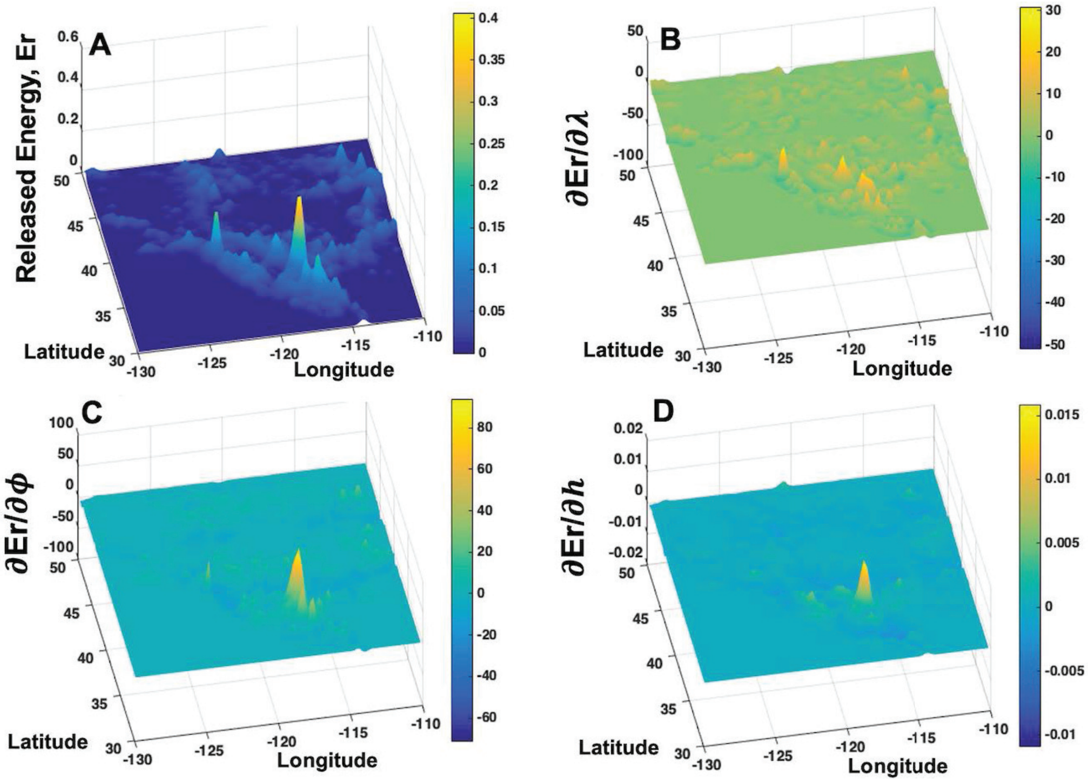

Fig. S11. Spatial Gradient of the pseudo released energy with respect to the Geodetic Coordinate System. **(A)** the pseudo released energy generated by the multiplicative combination of four exponential LFs with $(L, T), L = (12.5, 25)$ km and $T = (6, 12)$ epochs at the depth $z = 12.5$ km. **(B-D)** Spatial gradients of the pseudo released energy in the longitude (λ), latitude (ϕ), and depth (h) direction at depth 12.5 km downward, respectively. The increments of (0.2 deg, 0.2 deg, 5 km) are used for spatial discretization.

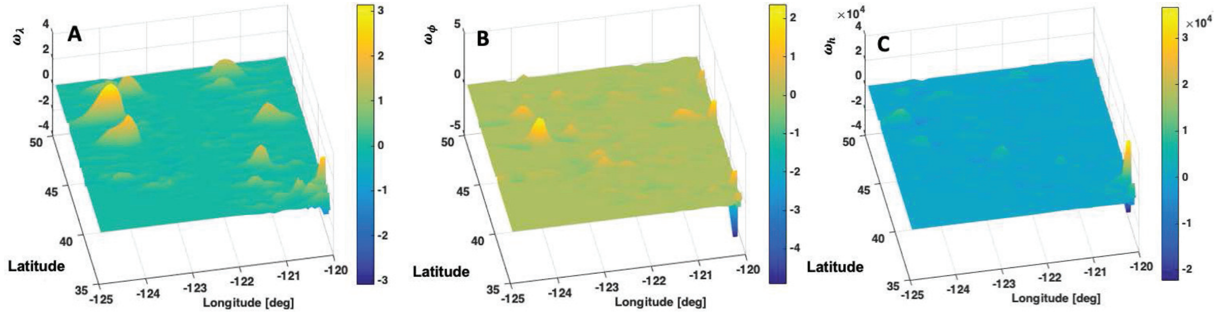

Fig. S12. Vorticity of the pseudo released energy flow at depth = 2.5 km generated from the past 10 years data (epochs from 10355 through 10473). **(A-C)** Three components of the vorticity calculated by Eq. (12).

$$A = \frac{L(EG - 2F^2) + 2EFM - E^2N}{2E(EG - F^2)} \tag{54}$$

$$B = \frac{EM - FL}{E\sqrt{EG - F^2}} \tag{55}$$

With notation of $\boldsymbol{X}_u = \partial \boldsymbol{X}/\partial u$ and $\boldsymbol{X}_{uu} = \partial^2 \boldsymbol{X}/\partial u^2$,

$$E = \boldsymbol{X}_u \cdot \boldsymbol{X}_u;\ F = \boldsymbol{X}_u \cdot \boldsymbol{X}_v;\ G = \boldsymbol{X}_v \cdot \boldsymbol{X}_v \tag{56}$$

$$\boldsymbol{N} = \frac{\boldsymbol{X}_u \times \boldsymbol{X}_v}{\|\boldsymbol{X}_u \times \boldsymbol{X}_v\|} \tag{57}$$

$$L = \boldsymbol{N} \cdot \boldsymbol{X}_{uu};\ M = \boldsymbol{N} \cdot \boldsymbol{X}_{uv};\ N = \boldsymbol{N} \cdot \boldsymbol{X}_{vv} \tag{58}$$

Note that the unit surface normal vector $\boldsymbol{N}$ is different from the constant N.

Then, from the surface $\boldsymbol{X}(u, v, Z)$ at a fixed depth h, we can calculate following derivatives at the point associated with the maximum value of Z.

$$\boldsymbol{X}_u = (1, 0, Z_u);\ \boldsymbol{X}_v = (0, 1, Z_v) \tag{59}$$

$$\boldsymbol{X}_{uu} = (0, 0, Z_{uu});\ \boldsymbol{X}_{vv} = (0, 0, Z_{vv});\ \boldsymbol{X}_{uv} = (0, 0, Z_{uv}) \tag{60}$$

$$E = 1 + Z_u^2;\ F = Z_u Z_v;\ G = 1 + Z_v^2 \tag{61}$$

$$\boldsymbol{N} = \frac{(-Z_u, -Z_v, 1)}{\sqrt{Z_u^2 + Z_v^2 + 1}} \tag{62}$$

$$L = \frac{Z_{uu}}{\sqrt{Z_u^2 + Z_v^2 + 1}};\ M = \frac{Z_{uv}}{\sqrt{Z_u^2 + Z_v^2 + 1}};\ N = \frac{Z_{vv}}{\sqrt{Z_u^2 + Z_v^2 + 1}} \tag{63}$$

For the derivative of the pseudo physics quantity Z, this study used the central difference method assuming the uniform internal in each direction.

$$Z_u \approx \frac{Z(u + \Delta u, v) - Z(u - \Delta u, v)}{2\Delta u} \tag{64}$$

$$Z_v \approx \frac{Z(u, v + \Delta v) - Z(u, v - \Delta v)}{2\Delta v} \tag{65}$$

$$Z_{uu} \approx \frac{Z(u + \Delta u, v) - 2Z(u, v) + Z(u - \Delta u, v)}{\Delta u^2} \tag{66}$$

$$Z_{vv} \approx \frac{Z(u, v + \Delta v) - 2Z(u, v) + Z(u, v - \Delta v)}{\Delta v^2} \tag{67}$$

$$Z_{uv} \approx \frac{Z(u + \Delta u, v + \Delta v) - Z(u + \Delta u, v - \Delta v) - Z(u - \Delta u, v + \Delta v) + Z(u - \Delta u, v - \Delta v)}{4\Delta u \Delta v} \tag{68}$$

Overall architecture of the Bayesian evolutionary algorithm for identifying the data-driven prediction model

The overall architecture of the Bayesian evolutionary algorithm framework developed for this study is summarized in Fig. S15. The central notion in Fig. S15 is in alignment with the author's recent successful applications to identification of hidden models behind nano-scale unknown phenomena (*32*) and complex heterogeneous structures (*33,34*). Compared to deep learn-ing, one of the central novelties is to "externalize" multi-layered convolutions by conducting multiple convolutions at the information level in Fig. S15A), not in the hidden layers or neu-rons. The starting point is raw data sets of earthquake hypocenters, of which spatial information is integrated via three-dimensional (3D) spatial convolution with multiple influence ranges (i.e. L_k, $k = 1$, ..., n_l). Then, the 3D convolved IIs are further integrated via temporal convolu-tions with multiple temporal influence ranges (T_l, $l = 1$, ..., n_T), thereby generating 4D spatio-temporal convolved IIs. Multiple convolved IIs and their interactions may be regarded as the counterparts to deep learning's multi-layered convolutions. Then, scientists' basic knowledge is infused into the diverse IIs to quantify the generic terms of energy, power, gradients, vorticity, or Laplacian (Fig. S15B). All these physics quantities are "pseudo" quantities since they are not from the first principle or direct physics theory. Still, they convey physical meanings. These basic quantities are derived from data via LFs and no other earthquake-related mechanisms. Thus, this approach pursues completely data-driven learning. Naturally, the inclusion of other physics concepts (e.g., heat, temperature, fluid) is straightforward as long as they are derived from observed data. Flexible and expressive LFs (Fig. S15C) identify mathematical expres-sions between IIs and the basic physics quantities in Fig. S15B. The revealed expressions will be about the imminent earthquake prediction as well as about physics quantities (Fig. S15D). The best-so-far expressions of the identified rules are remembered and reused as a prior best generation in Fig. S15D. Importantly, all the identified rules will hold clear interpretability,

enabling the expandability to other physics quantities. All the generated data sets (marked by the green cylinder in Fig. S15) are made publicly available upon request to the author.

Combination of the Bayesian update and evolutionary algorithm

Aiming at no distributional assumptions about the priors/posteriors as well as pursuing smooth evolution, this study adopts the combination of Bayesian update and a modified genetic algorithm. The key evolutionary algorithm involves the preparation of initial generation, organism-wise evaluation of fitness score, and fitness-based spawning of the next generation. The prior best physical rules can be smoothly inherited by the Bayesian update-based fitness proportionate probability (FPP) rule. To accelerate the evolution speed of the modified genetic algorithm, an individual variable-wise gene cross-over scheme has been used, and the changing search range scheme is used in an iterative manner for better performance as successfully done in (*32,33,34*). Since an individual s realizes a candidate of θ, all free parameters in current genera-tion S, the raw cost of an individual s, termed as $\mathcal{J}(s)$, is calculated by a number of types. In the definition of type, $M_{obs}^{(t+1)}(\boldsymbol{\xi}_j)$ means the observed maximum moment magnitude in the volume at epoch $(t+1)$ that is regarded as the true (measured) physical response. M_{thr} denotes the user-defined moment magnitude threshold. Then, following typical genetic algorithm procedure (*32, 33, 34*) the normalized fitness score $\mathcal{F}$ of an individual is calculated by

$$\mathcal{F}(s) = \frac{(1+\mathcal{J}(s))^{-1}}{\sum_{\forall s \in S}[(1+\mathcal{J}(s))^{-1}]} \tag{69}$$

where s denotes an individual in the entire generation S. Learning a hidden physical rule is not a one-time task, rather a continuous activity. As diverse new experimental data become available, the physical rule learner must embrace all the previous knowledge and learn new information. To seamlessly realize this continuous learning, this study infused the Bayesian update scheme into the evolutionary algorithm's FPP rule. Suppose we have the best-so-far generation, de-

noted as S^* and its associated fitness scores, $\mathcal{F}^*(s)$, $s \in S^*$. According to the FPP rule, the probability of selecting an θ for next parent is given by $\text{Prob}(\theta) \propto \mathcal{F}(s), s \in S^*$. Thus, $\mathcal{F}^*(s)$ is regarded as a prior PDF of parameters $\theta = \{\mathbf{a}, \mathbf{x}^*\}$, i.e. $\pi_{prior}(\theta)$ in the typical Bayesian formalism. For initialization of $\pi_{prior}(\theta)$, this study intentionally departs from fully random initialization to investigate positive evolution trends without special initialization assumption. Thus, this framework is purely data-driven, requiring no distributional assumptions about priors and posteriors. For the posterior distribution, we adopted the following two-stage procedure. Suppose that we have the prior best LFs and their S^* and that new experimental data become available. At the first learning generation with the new data, we can calculate the first fitness scores $\mathcal{F}(s; S^*)$ by applying the prior S^* and LFs to the new experiment. After the first generation, we can estimate the Bayesian fitness score (denoted as $\mathcal{F}_B$) as:

$$\mathcal{F}_B(s) = \frac{1}{\kappa} \frac{\mathcal{F}(s; S^*)\mathcal{F}^*(s)}{\sum_{\forall s \in S^*} \mathcal{F}^*(s)} \tag{70}$$

where κ is needed for normalizing the Bayesian fitness to unity, which is simply given by

$$\kappa = \sum_{\forall s \in S^*} \frac{\mathcal{F}(s; S^*)\mathcal{F}^*(s)}{\sum_{\forall s \in S^*} \mathcal{F}^*(s)} \tag{71}$$

Then, from the second learning generation of the new experiment, the probability of selecting two parents is proportional to the Bayesian fitness score as

$$\text{Prob}(\text{parent}_i | s) \propto \mathcal{F}_B(s), \ (i = 1, 2). \tag{72}$$

Once again, an individual s realizes a candidate of $\theta = (\mathbf{a}, \mathbf{x}^*)$ in the new generation S, and thus the desired posterior distribution is obtained. In this way, the prior knowledge is smoothly inherited to the new experiment on the framework of evolutionary algorithm, thereby enabling constantly evolving physical rule learning. For allowing for evolving with new data, the previous scores are inherited by the Bayesian score Eq. (70). Since the adopted evolutionary algorithm remembers prior generation's fitness scores, which offer the probability distribution

of free parameters of LFs. As the Bayesian inheritance continues with new experimental data, the probability distribution of LFs will naturally evolve. Thus, the proposed framework can achieve evolving capability with increasing data. In the future, more dedicated investigations should focus on validation of the constantly evolving capability of LFs with sufficient, diverse test data. To some extent, the combination of Bayesian update and evolutionary algorithm can be viewed as a log-likelihood maximization as explained in (*32,34*).

Holistic error measure specialized for the individual earthquake prediction

Another notable challenge of this study is rooted in the question of how to define an effective and efficient earthquake-specialized error (equivalently, loss or fitness). Sufficiently large earthquakes (e.g. moment magnitude $>$ 4.0) are not regarded as a point-wise phenomenon (e.g., rupture area $>$ 1 km^2), but rather they span certain spaces. A successful error measure for ML should be able to holistically quantify the discrepancy in magnitudes, locations, and false warnings of the predicted earthquakes. To fulfill such multifaceted objectives, this study proposed a comprehensive error measure. As explained in Fig. S16, a good prediction rule should be able to predict not only the magnitudes of future events but also their locations in the three-dimensional lithosphere with the smallest number of false predictions. Still, this study's error measure is open to improvement by incorporating further computational schemes and/or deeper physics-ingrained terms. In particular, this study proposes a holistic error function $\mathcal{J}$ in Eq. (73) that accommodates diverse errors in magnitude, location, and false alarms. The proposed error function places higher importance on the large events.

$$\mathcal{J}(s) = (1 - a_{cnt}) \sum_{\tilde{k} \in Top} \omega_{MD}^{(\tilde{k})} E_{MD}^{(\tilde{k})} / n(Top) + a_{cnt} E_{cnt} \tag{73}$$

$$E_{MD}^{(\tilde{k})} := a_M \text{erf}\left(\frac{|M_{obs}^{(t+1)}(\boldsymbol{\xi}_{\tilde{k}}) - M_{pred}^{(t+1)}(\boldsymbol{\xi}_{k^*})|}{M_{obs}^{(t+1)}(\boldsymbol{\xi}_{\tilde{k}})} \right) + (1 - a_M)\text{erf}\left(\frac{||\boldsymbol{\xi}_{\tilde{k}} - \boldsymbol{\xi}_{k^*}||_2}{r_{max}} \right) \tag{74}$$

$$E_{cnt} := \frac{1}{2}\text{erf}\left(\frac{|n(Top) - n(Top_{pred})|}{n(Top)}\right) + \frac{1}{2}\text{erf}\left(\sum_{\forall \tilde{k} \in Top_{pred}^{-1}} \frac{|M_{thr} - M_{pred}^{(t+1)}(\boldsymbol{\xi}_{\tilde{k}})|/M_{thr}}{n(Top_{pred}^{-1})}\right) \tag{75}$$

After sorting the observed real magnitudes in descending order, we can obtain Top, a set of indices of reference volumes that contains the sorted real magnitudes greater than M_{thr}, $Top := \{\tilde{k} \text{ of } \boldsymbol{\xi}_{\tilde{k}} |\ M_{obs}^{(t+1)}(\boldsymbol{\xi}_{\tilde{1}}) \geq M_{obs}^{(t+1)}(\boldsymbol{\xi}_{\tilde{2}}) \geq \cdots > M_{thr}\}$. Similarly, we obtain Top_{pred}, a set of indices of reference volumes that contains the sorted predicted magnitudes, $Top_{pred} := \{\tilde{k} \text{ of } \boldsymbol{\xi}_{\tilde{k}} |\ M_{pred}^{(t+1)}(\boldsymbol{\xi}_{\tilde{1}}) \geq M_{pred}^{(t+1)}(\boldsymbol{\xi}_{\tilde{2}}) \cdots > M_{thr}\}$. $k^*(\tilde{k}) \in Top_{pred}$ means the index of the spatially closest reference volume to $\tilde{k} \in Top$, obtained by $k^*(\tilde{k}) := \text{argmin}_{\forall k \in Top_{pred}} ||\boldsymbol{\xi}_{\tilde{k}} - \boldsymbol{\xi}_k||_2$. $E_{MD}^{(k)}$ in Eq. (74) considers a weighted average of errors in magnitude and location of earthquakes larger than the threshold M_{thr}. erf(.) is the Gauss error function used for mapping real-valued error $\in \mathbb{R}[0, \infty)$ to the range of [0,1] and its general definition is given by erf(z) $:= \frac{2}{\sqrt{\pi}} \int_0^z e^{-t^2} dt$ where $z \in \mathbb{C}$ and erf$(z) \in [-1, 1]$. A weight coefficient $a_M \in \mathbb{R}[0, 1]$ determines the relative importance of magnitude error compared to the location error. Here, $a_M = 0.5$ is used meaning the same importance in predicting magnitudes and locations. The proposed error function addresses the correct and false prediction of total events' count above the threshold by the second term of the right-hand side of Eq. (73). The relative importance of the counting error is weighted by $a_{cnt} \in \mathbb{R}[0, 1]$, herein $a_{cnt} = 0.1$ is used. $r_{max} = 200$ km is used to normalize the distance prediction error. Predictions only within r_{max} are considered in the error calculation while the predictions beyond r_{max} are regarded as incorrect predictions. Also, the proposed error measure puts increasing weights on the larger real earthquakes through $\omega_{MD}^{(\tilde{k})}$ defined as $\omega_{MD}^{(\tilde{k})} := \exp(M_{obs}^{(t+1)}(\boldsymbol{\xi}_{\tilde{k}})/10.0)$; $\tilde{k} \in Top$, which helps improve the accuracy of predicting larger, rare events. The additional error term E_{cnt} of Eq. (75) quantifies the wrong predictions since such "false alarm" may hamper reliability of the prediction and result in substantial societal cost. E_{cnt} consists of two terms, the first term is about how many false alarms happened in

terms of the total count while the second term is about how far the false alarms deviate from the minimum threshold M_{thr}. In Eq. (75) $Top_{pred}^{-1} := \{\tilde{k} \mid \tilde{k} \in Top_{pred}$ & never used in $E_{MD}^{(\tilde{k})}\}$.

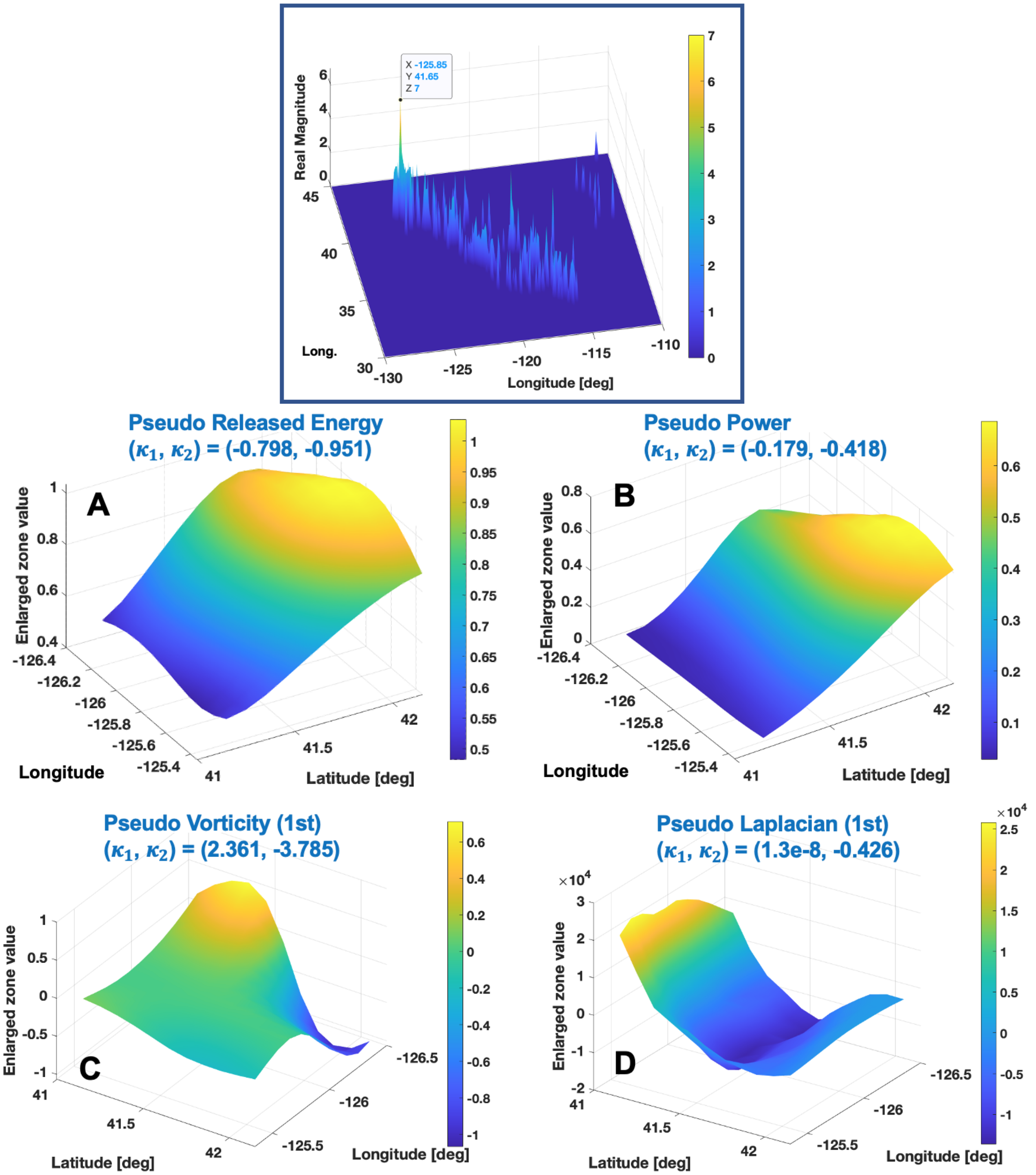

Fig. S13. Example plots of pseudo physics quantities generated with 10 years data up to one month before the epoch 10139: (A-D) Enlarged plots of the pseudo released energy (E_r), the pseudo power ($\partial E_r/\partial t$), and the first terms of pseudo vorticity ω_λ and Laplacian $\partial^2 E_r/\partial \lambda^2$ near the peak ($M_w = 7$) zone covering ($-125.85° \pm 1°, 41.65° \pm 1°$) at depth 2.5 km. The calculated principal Gauss curvatures (κ_1, κ_2) are shown. Top inset shows the real earthquakes distribution including the peak. Axes of A-D are intentionally arranged to better show the surface curvatures–concave, convex, or flat.

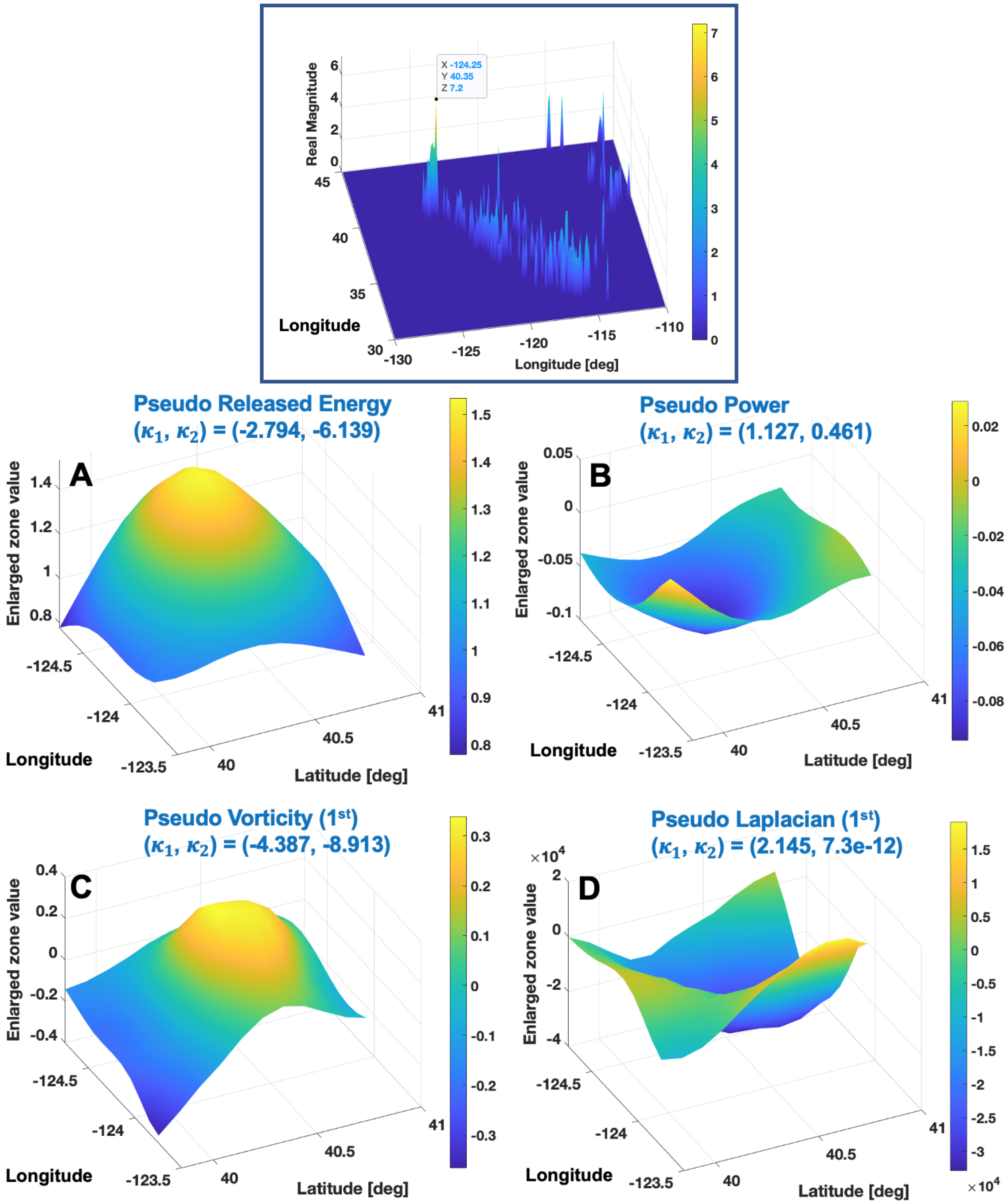

Fig. S14. Example plots of pseudo physics quantities generated with 10 years data up to one month before the epoch 10147: (A-D) Enlarged plots of the pseudo released energy (E_r), the pseudo power ($\partial E_r/\partial t$), the first term of pseudo vorticity ω_λ, and the first term of the pseudo Laplacian $\partial^2 E_r/\partial \lambda^2$ near the peak ($M_w = 7.2$) zone covering $(-124.25° \pm 1°, 40.35° \pm 1°)$ at depth 7.5 km. The calculated principal Gauss curvatures (κ_1, κ_2) are shown. Top inset shows the real earthquakes distribution including the peak.

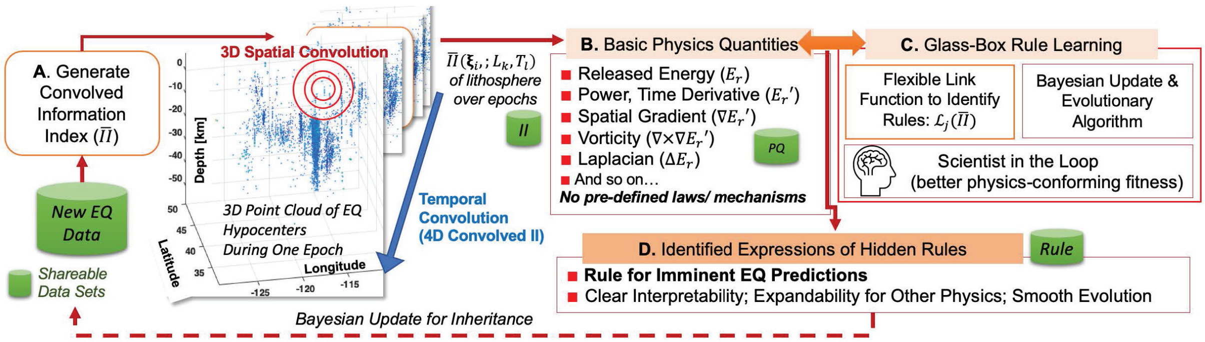

Fig. S15. Overall architecture of the Bayesian evolutionary algorithm framework for identifying individual earthquake prediction model. **(A)** Externalized information convolution to generate spatio-temporal 4D convolved information index. **(B)** Data-driven basic physics (pseudo) quantities, not driven by pre-defined earthquake-related mechanisms. **(C)** Rule learning core using flexible link functions (LFs), a combination of Bayesian update and evolutionary algorithm, and scientist-in-the-loop for infusing scientists' knowledge into fitness (error) measures. **(D)** Remember the best-so-far expressions of identified rules. Shareable data sets are for other ML methods applications.

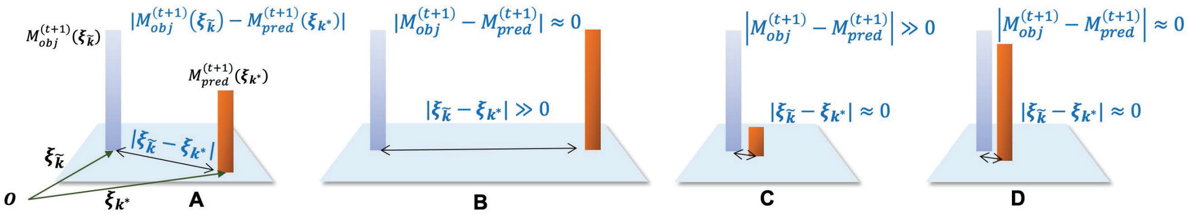

Fig. S16. Illustration of Comprehensive Error Calculation. **(A)** Definition of M_{obj} and M_{pred}, the observed and predicted moment magnitudes, respectively, which are closest each other. Examples of poor predictions due to the location error **(B)** or due to the the magnitude error **(C)**. Good prediction of both magnitude and location **(D)**.
